# The Nesprin Family Member ANC-1 Regulates Synapse Formation and Axon Termination by Functioning in a Pathway with RPM-1 and β-Catenin

**DOI:** 10.1371/journal.pgen.1004481

**Published:** 2014-07-10

**Authors:** Erik D. Tulgren, Shane M. Turgeon, Karla J. Opperman, Brock Grill

**Affiliations:** 1 Department of Pharmacology, University of Minnesota, Minneapolis, Minnesota, United States of America; 2 Department of Neuroscience, The Scripps Research Institute - Florida, Jupiter, Florida, United States of America; Howard Hughes Medical Institute, United States of America

## Abstract

Mutations in Nesprin-1 and 2 (also called Syne-1 and 2) are associated with numerous diseases including autism, cerebellar ataxia, cancer, and Emery-Dreifuss muscular dystrophy. Nesprin-1 and 2 have conserved orthologs in flies and worms called MSP-300 and abnormal nuclear Anchorage 1 (ANC-1), respectively. The Nesprin protein family mediates nuclear and organelle anchorage and positioning. In the nervous system, the only known function of Nesprin-1 and 2 is in regulation of neurogenesis and neural migration. It remains unclear if Nesprin-1 and 2 regulate other functions in neurons. Using a proteomic approach in *C. elegans*, we have found that ANC-1 binds to the Regulator of Presynaptic Morphology 1 (RPM-1). RPM-1 is part of a conserved family of signaling molecules called Pam/Highwire/RPM-1 (PHR) proteins that are important regulators of neuronal development. We have found that ANC-1, like RPM-1, regulates axon termination and synapse formation. Our genetic analysis indicates that ANC-1 functions via the β-catenin BAR-1, and the ANC-1/BAR-1 pathway functions cell autonomously, downstream of RPM-1 to regulate neuronal development. Further, ANC-1 binding to the nucleus is required for its function in axon termination and synapse formation. We identify variable roles for four different Wnts (LIN-44, EGL-20, CWN-1 and CWN-2) that function through BAR-1 to regulate axon termination. Our study highlights an emerging, broad role for ANC-1 in neuronal development, and unveils a new and unexpected mechanism by which RPM-1 functions.

## Author Summary

The molecular mechanisms that underpin synapse formation and axon termination are central to forming a functional, fully connected nervous system. The PHR proteins are important regulators of neuronal development that function in axon outgrowth and termination, as well as synapse formation. Here we describe the discovery of a novel, conserved pathway that is positively regulated by the *C. elegans* PHR protein, RPM-1. This pathway is composed of RPM-1, ANC-1 (a Nesprin family protein), and BAR-1 (a canonical β-catenin). Nesprins, such as ANC-1, regulate nuclear anchorage and positioning in multinuclear cells. We now show that in neurons, ANC-1 regulates neuronal development by positively regulating BAR-1. Thus, Nesprins are multi-functional proteins that act through β-catenin to regulate neuronal development, and link the nucleus to the actin cytoskeleton in order to mediate nuclear anchorage and positioning in multi-nuclear cells.

## Introduction

The mammalian Nuclear Envelope Spectrin repeat proteins (Nesprins) (also called Syne-1/Enaptin and Syne-2/NUANCE) mediate the anchorage of nuclei in multinucleated cells such as muscle [1], [2], and mediate nuclear movement and positioning in mononuclear cells [3], [4]. The orthologs of Nesprin-1 and 2 are called MSP-300 in Drosophila and abnormal nuclear Anchorage 1 (ANC-1) in *C. elegans*. MSP-300 and ANC-1 also function in nuclear anchorage, and regulate positioning of organelles including mitochondria and the endoplasmic reticulum [5], [6].

Nesprin family members are attached to the nuclear envelope by the SUN proteins (SUN 1 and 2), which together compose the Linker of the Nucleoskeleton and Cytoskeleton (LINC) complex [1], [6]. A C-terminal Klarsicht/ANC-1/Nesprin homology (KASH) domain anchors Nesprin-1 and 2 in the outer nuclear membrane by binding to SUN1 and 2, which are localized to the inner nuclear membrane. *C. elegans* has two SUN family proteins: Uncoordinated 84 (UNC-84) (retains ANC-1 in the nuclear membrane; expressed in most somatic cells) and SUN-1 (functions in the germ line and early embryo). Tandem calponin homology domains at the N-terminus of the Nesprins mediate binding to the actin cytoskeleton.

Nesprin-1 and 2 have functions outside of their role in nuclear anchorage. Nesprin-1 and 2 regulate centrosome orientation in migrating cells and ciliogenesis [3], [4] and regulate formation and trafficking of the Golgi [7]. Importantly, mutations in Nesprin-1 and 2 are associated with numerous diseases including: autism [8], [9], cerebellar ataxia [10], Emery Dreifuss muscular dystrophy [11], cancer [12], arthrogryposis [13], and cardiomyopathy [14]. Genome-wide association studies have also identified single nucleotide polymorphisms in Nesprin-1 that are associated with schizophrenia [15] and bipolar disorder [16], [17].

Nesprin-1 and 2 perform several functions at the neuromuscular junction (NMJ) and in the central nervous system (CNS). At the NMJ, multiple nuclei are anchored in clusters directly adjacent to the postsynaptic terminal. Nesprin-1 is enriched on these postsynaptic nuclei [18], and required for their clustering [19]. Nesprin-1 is required for axon termination of motor neurons that innervate the diaphragm [2]. Nesprin-1 and 2 are also expressed in neurons of the CNS [18], where they function in neuronal migration and neurogenesis by mediating connections between the nucleus and the cytoskeleton [4], [20]. Of particular note, Nesprin-1 shows extremely strong, broad expression in the adult murine CNS, which suggests that Nesprin-1 is likely to have an important function in neurons beyond the role it plays in neural precursor migration and neurogenesis (Allen Brain Atlas: http://mouse.brain-map.org) [21]. This is consistent with the observation that an extremely small splice variant of Nesprin-1 called Candidate Plasticity Gene 2 (CPG2) regulates synaptic plasticity [22]. At present, it remains unclear if Nesprin family members play broader roles in neuronal function and development outside of their roles in very early developmental events such as neurogenesis, and neural migration.

The role of Nesprin-1 and 2 in signal transduction has begun to be explored, but remains relatively poorly understood. In vascular smooth muscle cells, small isoforms of Nesprin-2 regulate Erk MAP kinase signaling [23]. Studies using a keratinocyte cell line showed that Nesprin-2 binds to α- and β-catenin, and regulates the nuclear localization of β-catenin [24]. While these studies demonstrate that Nesprin-2 has the potential to regulate signal transduction, the broader functional consequences of these activities remain unclear. Further, it remains unknown if Nesprin-1 and/or Nesprin-2 mediate signal transduction in neurons.

Members of the Pam/Highwire/RPM-1 (PHR) protein family are large signaling proteins that include: human Pam (also called MYCBP2), murine Phr1, zebrafish Phr1 (Esrom), Drosophila Highwire, and *C. elegans* Regulator of Presynaptic Morphology 1 (RPM-1) [25]. The PHR proteins are important regulators of neuronal development that function in axon outgrowth and termination [26]–[28], axon guidance [29]–[31], and synapse formation [32]–[34]. PHR proteins also function in axon regeneration [35], [36] and axon degeneration following damage [37], [38].

The PHR proteins regulate several conserved signal transduction pathways [39]–[45]. However, it is poorly understood if PHR protein activity is linked to signaling by extracellular guidance cues, morphogens, or adhesion molecules. Work in Drosophila and *C. elegans* has shown that Highwire negatively regulates BMP signaling [46], and RPM-1 negatively regulates Slit and Netrin signaling [29]. However, it remains unclear if PHR protein activity converges with extracellular cues on common signaling targets. It is also uncertain if the PHR proteins have the ability to positively regulate, modify or enhance signals generated by extracellular cues.

Using a proteomic approach in *C. elegans*, we have identified ANC-1 as an RPM-1 binding protein. Similar to *rpm-1*, *anc-1* functions in both axon termination and synapse formation. Our analysis indicates that *anc-1* functions in a genetic pathway with *beta-catenin/armadillo related protein 1 (bar-1)* downstream of *rpm-1*. Further, we identify the Wnt signaling mechanisms that regulate BAR-1 to control axon termination. Our observations provide the first evidence of a link between RPM-1 signaling and the Wnt ligands that regulate neuronal development.

## Results

### Identification of ANC-1 as an RPM-1 binding protein

To better understand the mechanism of how RPM-1 functions in neuronal development, we previously performed a proteomic screen to identify RPM-1 binding proteins [42]. Briefly, RPM-1 fused with Green Fluorescent Protein (GFP) was transgenically expressed using the native *rpm-1* promoter. This construct was purified from whole worm lysate using an anti-GFP antibody, and RPM-1 binding proteins were identified using mass spectrometry and *de novo* peptide sequencing. To date, our screen has successfully identified three functional RPM-1 binding proteins: Gut Granule Loss (GLO-4, a putative Rab GEF) [42], RNA Export factor 1 (RAE-1, a microtubule binding protein) [43], and Protein Phosphatase Mg^2+^/Mn^2+^ dependent 2 (PPM-2, a PP2C phosphatase) [47]. Our screen also identified the F-box Synaptic Protein 1 (FSN-1) [42], which was previously discovered using a genetic approach [45]. Importantly, GLO-4, RAE-1, and PPM-2 are not targets of RPM-1 ubiquitin ligase activity. Thus, our proteomic screen preferentially identified RPM-1 binding proteins that are not degraded by RPM-1, and are stable interaction partners.

**Figure 1:**
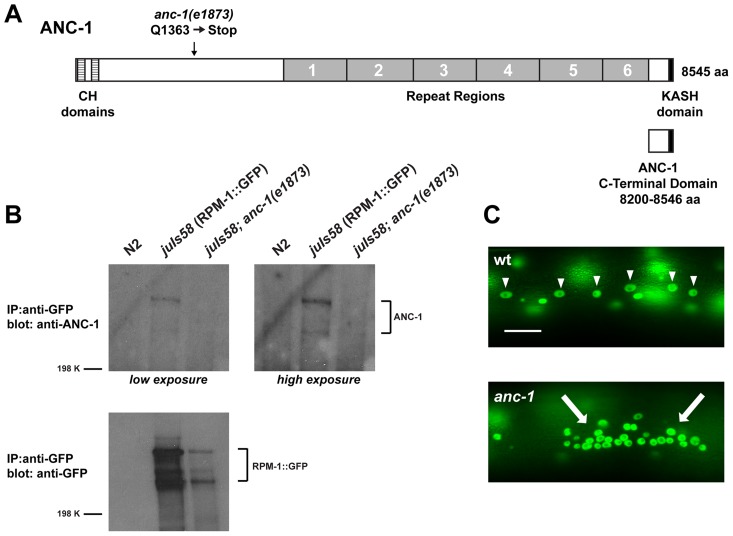
ANC-1 binds to RPM-1. (A) Schematic of ANC-1 protein structure which consists of two calponin homology (CH) domains that bind F-actin (dashed boxes), 6 repeat regions (grey), and a KASH domain (black) that mediates binding to the nucleus. Also shown is the C-terminal domain of ANC-1 that functions as a dominant negative. (B) CoIP of endogenous ANC-1 with RPM-1::GFP. CoIPs were performed from whole worm lysates prepared from transgenic animals (*juIs58*) or non-transgenic animals (N2). (C) Epifluorescent microscopy was used to visualize SUR-5::GFP in the multinucleated hypodermal cells of *C. elegans*. In wild-type animals, nuclei are anchored to the actin cytoskeleton and evenly spaced throughout the syncytium (arrowheads). In *anc-1* mutants, impaired nuclear anchorage leads to aggregation of nuclei (arrows). Scale bar is 20 µm.

Another RPM-1 binding protein identified in our proteomic screen was ANC-1, a gigantic protein that is composed of 8545 amino acids and has an approximate molecular weight of 956 kDa. ANC-1 consists mostly of predicted coiled regions (including six repeats that are nearly identical at the nucleotide level), two N-terminal calponin-homology (CH) domains that bind to actin, and a C-terminal KASH domain that targets ANC-1 to the nuclear envelope (Figure 1A). Previous work showed that ANC-1 is present at the nuclear envelope and in the cytoplasm of all post-embryotic somatic cells [6].

Our proteomic analysis identified 10 peptides that covered 6.3% of the total ANC-1 protein sequence (Figure S1). The majority of peptide sequence identified was from the ANC-1 specific repeats, presumably because repeat sequence is present at 6-fold molar excess over other regions of ANC-1. To confirm the biochemical interaction between ANC-1 and RPM-1 we utilized coimmunoprecipitation (coIP) from whole worm lysates generated from transgenic animals. A prior study developed polyclonal anti-ANC-1 antibodies that recognize endogenous ANC-1, which was detected as multiple high molecular weight bands in immunoblots [6]. We used these anti-ANC-1 antibodies in coIP experiments with transgenic animals expressing RPM-1::GFP (*juIs58*). When RPM-1::GFP was immunoprecipitated using an anti-GFP antibody, coprecipitating ANC-1 was detected as multiple high molecular weight bands (Figure 1B, *juIs58*). Further examples of this coIP are shown in Figure S2. Coprecipitating bands were absent or strongly reduced in intensity in *juIs58; anc-1* mutants demonstrating that these bands represent endogenous ANC-1 (Figure 1B). Coprecipitating ANC-1 was not detected in precipitates from non-transgenic animals (Figure 1B, N2). Thus, ANC-1 did not bind non-specifically to the agarose beads or the anti-GFP antibody, which demonstrates that the interaction between ANC-1 and RPM-1 is specific. These biochemical results confirm that RPM-1 binds to ANC-1, or a protein complex that contains ANC-1.

### *anc-1* regulates synapse formation in the GABAergic motor neurons

**Figure 2:**
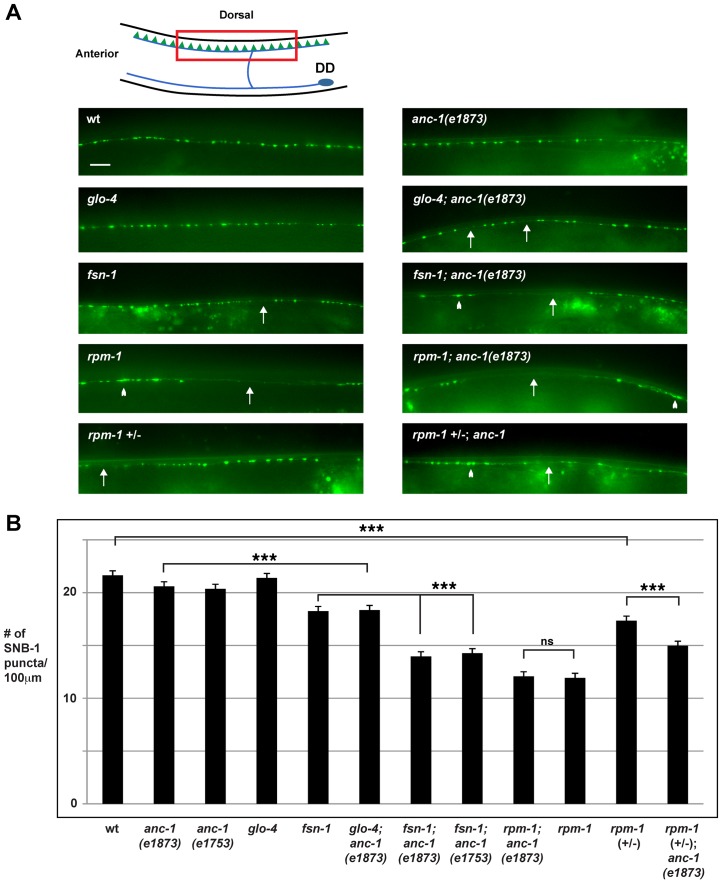
*anc-1* regulates synapse formation in the GABAergic motor neurons. (A) Upper panel diagrams the GABAergic DD neurons (blue) that innervate dorsal muscles (inspired by Worm Atlas). DD Presynaptic terminals are shown in green. The red box highlights the region of the dorsal cord that was visualized by epifluorescent microscopy. The transgene *juIs1* [P_unc-25_SNB-1::GFP] was used to visualize the presynaptic terminals for the indicated genotypes. Arrows highlight regions lacking presynaptic terminals represented by SNB-1::GFP puncta. Arrowheads note abnormal aggregation of presynaptic terminals. Scale bar is 10 µm. (B) Quantitation of the average number of SNB-1::GFP puncta per 100 µm of dorsal cord for the indicated genotypes. Analysis was done on young adults grown at 25°C. Significance was determined using an unpaired Student's *t* test; error bars represent the standard error of the mean. ***P<0.001, ns = not significant.

Previous studies have shown that *rpm-1* regulates synapse formation in the GABAergic dorsal D (DD) motor neurons [32]. The DD motor neurons innervate, and inhibit the dorsal muscles of the worm (Figure 2A, schematic). The presynaptic terminals of DD neurons can be visualized in living animals using the transgene *juIs1*, which uses a cell specific promoter (*Punc-25*) to express a fusion protein of Synaptobrevin-1 and GFP (SNB-1::GFP) [48]. In wild type animals, SNB-1::GFP localized to evenly sized puncta that were uniformly positioned along the dorsal nerve cord (Figure 2A). In *rpm-1(ju44)* mutants, SNB-1::GFP puncta in the dorsal nerve cord were abnormally aggregated (Figure 2A, arrowheads), and there were regions of the cord lacking any puncta (Figure 2A, arrows). Quantitation showed that the number of SNB-1::GFP puncta in *rpm-1* mutants was significantly lower than wild-type animals (compare 11.9±0.4 SNB-1::GFP puncta/100 µm for *rpm-1* with 21.9±0.4 puncta/100 µm for wild type, Figure 2B). These findings are consistent with results from previous studies [32]. Importantly, previous electron microscopy studies showed that the defects in SNB-1::GFP puncta localization in *rpm-1* mutants reflect defects in synapse formation, rather than defects in the formation of presynaptic terminals or the trafficking of synaptic vesicles [32], [39]. Subsequent studies have also shown that milder defects in organization of SNB-1::GFP puncta, such as those that occur in *fsn-1* mutants, are also due to defects in both pre and postsynaptic terminals [49].

Our observation that ANC-1 binds to RPM-1 led us to hypothesize that *anc-1* might function in synapse formation similar to *rpm-1*. To test this hypothesis, we analyzed two alleles of *anc-1*, *e1873* and *e1753*. DNA sequencing confirmed that *e1873* is a nonsense mutation that results in a severely truncated protein (Figure 1A) [6]. The molecular nature of the lesion in *e1753* remains unknown. However, a previous study showed that antibodies raised against the repeat region of ANC-1 fail to detect all isoforms of ANC-1 greater than 175 kDa in both *anc-1(e1873)* and *anc-1(e1753)* mutants [6]. Thus, *e1873* and *e1753* are likely to be molecular null alleles of *anc-1*.

Previous studies have shown that *anc-1* loss of function (lf) results in abnormal nuclear anchorage (Anc phenotype) of the nuclei in the syncytial cells that form the hypodermis of *C. elegans* [6]. The hypodermal nuclei can be visualized using a transgene, *kuIs54*, which expresses Suppressor of Activated LET-60 Ras (SUR-5)::GFP [50]. In wild-type animals, the hypodermal nuclei are anchored to the cytoskeleton, and distributed in a well organized, even pattern (Figure 1C, arrowheads). Consistent with previous results, we observed that *anc-1* mutants display an Anc phenotype, in which the nuclei are no longer anchored and aggregate dramatically (Figure 1C, arrows).

With regard to synapse formation, *anc-1* mutants had normal spatial distribution and number of SNB-1::GFP puncta (Figure 2A and B). We also constructed double mutants of *anc-1* with two members of the RPM-1 pathway, *fsn-1* and *glo-4*. FSN-1 is an F-box protein that binds to RPM-1 and mediates RPM-1 ubiquitin ligase activity [45]. GLO-4 binds to RPM-1, and is a putative guanine nucleotide exchange factor for a Rab pathway [42]. We found that *fsn-1; anc-1* and *glo-4; anc-1* double mutants had enhanced defects in synapse formation compared to single mutants (compare 14.0±0.5 puncta/100 µm for *fsn-1; anc-1(e1873)* with 18.3±0.3 for *fsn-1*, Figure 2A and B). *rpm-1; anc-1* double mutants had similar defects to those observed in *rpm-1* single mutants (Figure 2A and B). Additionally, we constructed *rpm-1 (+/−); anc-1* animals and found enhanced defects in synapse formation compared to *rpm-1 (+/−)* animals (Figure 2A and B). These results are consistent with several conclusions. First, because we used null alleles, we conclude that *anc-1* functions in a parallel genetic pathway to both *fsn-1* and *glo-4* to regulate synapse formation. Second, our observation that *rpm-1; anc-1* double mutants were not enhanced, in a phenotypic assay that is not saturated [45], suggests that *anc-1* functions in the same genetic pathway as *rpm-1*. This conclusion is further supported by our observation that *rpm-1 (+/−); anc-1* animals had enhanced defects in synapse formation. Third, these results support the conclusion that ANC-1 is not negatively regulated by RPM-1 and, therefore, is unlikely to be a target of RPM-1 ubiquitin ligase activity. If this were the case, we would expect to see suppression of synapse formation defects in *anc-1; rpm-1* double mutants similar to what was shown for Dual Leucine Zipper-bearing Kinase 1 (DLK-1), a known target of RPM-1 ubiquitin ligase activity [39].

### The β-catenin *bar-1* functions in the same genetic pathway as *anc-1* to regulate synapse formation

We next sought to dissect the mechanism of how *anc-1* regulates synapse formation. A previous study found that Nesprin-2 (a mammalian ortholog of ANC-1) regulates nuclear localization of β-catenin, thereby potentially regulating canonical Wnt signaling [24]. When Wnt signaling is not active, β-catenin is degraded; when Wnt signaling is activated, β-catenin accumulates, enters the nucleus, and interacts with T Cell Specific Transcription Factor (TCF)/Lymphoid Enhancer Binding Factor (LEF) family transcription factors to promote gene expression [51]. Previous work in *C. elegans* has shown that Wnt signaling regulates neuronal development [52]–[54]. In addition, Abnormal Cell Lineage 23 (LIN-23), an F-box protein that negatively regulates β-catenin in *C. elegans*, regulates the abundance of postsynaptic glutamate receptors in the ventral nerve cord [55], as well as axon termination in mechanosensory neurons [56]. Both of these developmental events are also regulated by *rpm-1* [27], [57]. Based on this prior work, we hypothesized that *anc-1* may function as a genetic link between *rpm-1* and β-catenin signaling.

**Figure 3:**
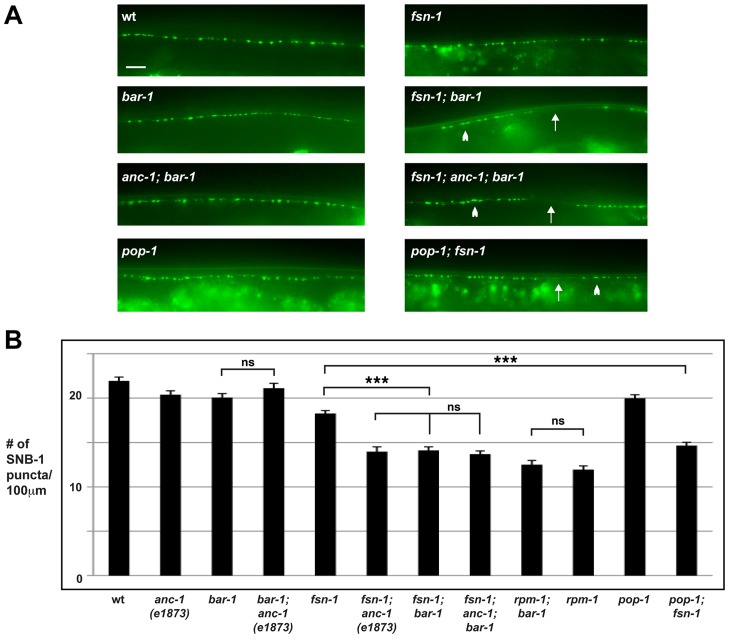
*anc-1* and *bar-1* function in the same genetic pathway to regulate synapse formation. (A) Epifluorescent microscopy was used to visualize presynaptic terminals labeled using the transgene *juIs1* for the indicated genotypes. Arrows highlight regions lacking presynaptic terminals represented by SNB-1::GFP puncta. Arrowheads note abnormal aggregation of presynaptic terminals. Scale bar is 10 µm. (B) Quantitation of the average number of SNB-1::GFP puncta per 100 µm of dorsal cord for the indicated genotypes. Analysis was done on young adults grown at 25°C. Significance was determined using an unpaired Student's *t* test; error bars represent the standard error of the mean. ***P<0.001, ns = not significant.

To test this hypothesis, we started by determining if β-catenin regulates synapse formation in the DD motor neurons. In *C. elegans*, there are four β-catenins that have diverged to perform separate functions [58], [59]. The canonical Wnt pathway operates through the β-catenin homolog BAR-1 and a single TCF homolog, Posterior Pharynx Defect 1 (POP-1) [60]. To test the role of *bar-1* in synapse formation, we analyzed a null allele of *bar-1, ga80* [61]. Consistent with a prior study, we observed that small, consistent sections of the dorsal cord were absent in *bar-1* (lf) mutants (data not shown) [62]. However, we were able to analyze synapse formation in sections of the dorsal cord that formed normally. *bar-1* mutants showed a distribution and number of SNB-1::GFP puncta that were similar to wild-type animals (Figure 3A and B). In contrast, *fsn-1; bar-1* double mutants showed enhanced defects in synapse formation (compare 14.1±0.4 SNB-1::GFP puncta/100 µm for *fsn-1; bar-1* with 18.3±0.3 puncta/100 µm for *fsn-1*, Figure 3A and B). The enhanced phenotype in *fsn-1; bar-1* double mutants was similar to what was observed for *fsn-1; anc-1* double mutants (Figure 3B). This result suggested that *anc-1* and *bar-1* may function in the same genetic pathway. To test this possibility, we constructed double and triple mutants between *bar-1*, *anc-1* and *fsn-1*. *anc-1; bar-1* double mutants were not enhanced compared to *bar-1* and *anc-1* single mutants (Figure 3A and B). Likewise, *fsn-1; anc-1; bar-1* triple mutants were not enhanced compared to *fsn-1; bar-1* or *fsn-1; anc-1* double mutants (Figure 3A and B).

To determine if BAR-1 regulates synapse formation by acting through a canonical signaling pathway that includes the TCF transcription factor POP-1, we analyzed the role of *pop-1* in synapse formation. Because null alleles of *pop-1* are lethal, we opted to analyze a hypomorphic allele of *pop-1, q645*. The POP-1 protein produced by *q645* shows reduced interaction with BAR-1 due to a mutation in the β-catenin binding domain [63]. Similar to our findings with *bar-1*, *pop-1* (lf) animals were largely wild type, but *pop-1; fsn-1* double mutants had enhanced defects in synapse formation that were of similar severity to *fsn-1; bar-1* double mutants (compare 14.6±0.4 SNB-1::GFP puncta/100 µm for *pop-1; fsn-1* with 18.2±0.3 puncta/100 µm for *fsn-1*, Figure 3A and B).

To determine if *bar-1* functions in the same pathway as *rpm-1*, we constructed *bar-1; rpm-1* double mutants. We observed no change in the severity of synapse formation defects in *bar-1; rpm-1* double mutants compared to *rpm-1* single mutants (Figure 3B).

As a whole, our results support several conclusions. First, *anc-1*, *bar-1* and *rpm-1* function in the same genetic pathway to regulate synapse formation. Second, the *anc-1/bar-1* pathway acts in parallel to *fsn-1* to regulate synapse formation. Finally, *bar-1* is likely to regulate synapse formation by functioning through a canonical Wnt signaling pathway that includes the TCF transcription factor *pop-1*.

### *anc-1* regulates axon termination in the mechanosensory neurons

Previous work showed that *rpm-1* regulates axon termination in the mechanosensory neurons that sense soft touch [27]. The posterior lateral microtubule (PLM) and the anterior lateral microtubule (ALM) mechanosensory neurons are an excellent system in which to study axon termination. Each PLM and ALM neuron extends a single axon that terminates extension at a precise anatomical location [64]. In addition, these neurons are easily visualized using a transgene (*muIs32*) that expresses GFP specifically in the mechanosensory neurons [65].

**Figure 4:**
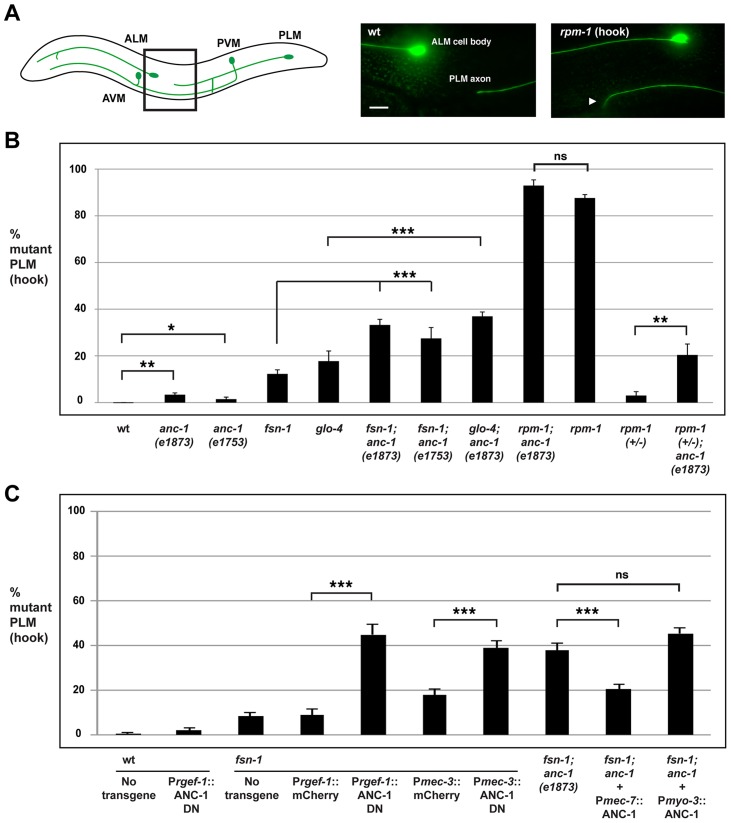
*anc-1* functions cell autonomously to regulate axon termination in the PLM mechanosensory neurons. (A) Upper panel diagrams the mechanosensory neurons of *C. elegans* (inspired by Worm Atlas). PLM neurons were visualized using *muIs32* [P_mec7_GFP]. The black box indicates the region of the animal that is visualized by epifluorescent microscopy and shown on the right. Shown for the *rpm-1* mutant is the PLM axon termination phenotype that we refer to as a hook defect (arrowhead). Scale bar is 10 µm. (B) Quantitation of axon termination (hook) defects in PLM neurons for the indicated genotypes. (C) An ANC-1 dominant negative construct (ANC-1 DN) was expressed using a pan-neuronal promoter (P*rgef-1*) or a mechanosensory neuron specific promoter (P*mec-3*) with the indicated genotypes. A full length ANC-1 rescue construct (P*mec-7*::ANC-1) was expressed in *anc-1; fsn-1* double mutants. The data shown is an average of 5 or more transgenic lines for each genotype. Analysis was done on young adults grown at 23°C. Significance was determined using an unpaired Student's *t* test; error bars represent the standard error of the mean. *P<0.05, **P<0.01, ***P<0.001, ns = not significant.

*C. elegans* contains two PLM neurons that sense soft touch in the posterior of the animal's body. Each PLM neuron has a single axon that terminates extension prior to the cell body of the ALM neuron (Figure 4A, schematic). In *rpm-1* (lf) mutants, PLM axons fail to terminate extension properly, grow past the ALM cell body and hook towards the ventral side of the animal (Figure 4A) [27], [42]. This defect, which we refer to as a “hook”, is highly penetrant in *rpm-1* mutants (87.6±1.5%, Figure 4B). Likewise, *rpm-1* mutants have highly penetrant axon termination defects in the ALM neurons (Figure S3A) [27], [42].

To determine whether *anc-1* also functions in axon termination, we analyzed *anc-1* animals as well as double mutants of *anc-1* and members of the *rpm-1* signaling pathway. Both *anc-1(e1873)* and *anc-1(e1753)* showed PLM hook defects that were significant compared to wild-type animals, but occurred with extremely low penetrance compared to *rpm-1* mutants (3.4±0.8% for *anc-1(e1873)* and 1.9±0.8% for *anc-1(e1753)*, Figure 4B). *fsn-1; anc-1* double mutants showed enhanced penetrance of defects compared to single mutants (compare 33.2±2.4 for *fsn-1; anc-1(e1873)* and 27.5±4.6% for *fsn-1; anc-1(e1753)* with 9.1±1.1% for *fsn-1*, Figure 4B). Likewise, *glo-4; anc-1* double mutants also showed enhanced penetrance of defects compared to single mutants (compare 36.9±1.9% for *glo-4; anc-1(e1873)* with 17.7±4.3% for *glo-4*, Figure 4B). We observed similar results when the function of *anc-1* was analyzed with regard to axon termination in the ALM neurons (Figure S3A). These results demonstrate that *anc-1* regulates axon termination by functioning in a parallel pathway to both *fsn-1* and *glo-4*.

We also analyzed *rpm-1; anc-1* double mutants, which had the same penetrance of defects as *rpm-1* single mutants. This result suggests that *anc-1* functions in the same genetic pathway as *rpm-1* (Figure 4B). Consistent with this conclusion, we observed enhanced axon termination defects in both the PLM and the ALM neurons of *rpm-1 (+/−); anc-1* animals (Figure 4B and Figure S3A).

Having established that *anc-1* regulates axon termination by functioning in the same pathway as *rpm-1*, we wanted to test if *anc-1* functions cell autonomously in the PLM neurons to regulate axon termination. Due to the enormous size of the *anc-1* gene, our first approach was to transgenically overexpress a dominant negative fragment of ANC-1. Previous work by Starr and Han showed that a C-terminal fragment of ANC-1 that contains the KASH domain (see Figure 1A, C-terminal domain aa 8200–8546) acts as a dominant negative by blocking binding of endogenous ANC-1 to the SUN domain protein UNC-84, thereby preventing ANC-1 localization to the nuclear envelope [6]. We engineered *fsn-1* mutants with transgenic extrachromosomal arrays that used a strong, pan-neuronal promoter (P*rgef-1*) to overexpress either the dominant negative ANC-1, or mCherry as a negative control. We observed enhanced penetrance of axon termination defects in *fsn-1* mutants expressing the ANC-1 dominant negative (44.9±4.6%) compared to animals expressing mCherry (8.9±2.6%, Figure 4C). Transgenic overexpression of dominant negative ANC-1 in wild-type animals did not give a significant phenotype (Figure 4C). These results are consistent with ANC-1 functioning in neurons to regulate axon termination.

To more directly address cell autonomy, we used a cell specific promoter, P*mec-3*, to transgenically overexpress the ANC-1 dominant negative specifically in the mechanosensory neurons. In *fsn-1* mutants, when the *mec-3* promoter was used to overexpress the ANC-1 dominant negative we observed enhanced penetrance of axon termination defects (38.8±3.3%) compared to *fsn-1* mutants that transgenically overexpressed mCherry (17.9±2.5%, Figure 4C). Thus, transgenic overexpression of dominant negative ANC-1, specifically in the mechanosensory neurons, enhances *fsn-1* (lf) to levels that are similar to what we observed in *fsn-1; anc-1* double mutants (Figure 4C).

To provide further evidence that *anc-1* functions cell autonomously in the mechanosensory neurons, we generated *fsn-1; anc-1* double mutants that carried a transgenic extrachromosomal array containing an *anc-1* mini-gene and a promoter, P*mec-7*, that drives expression specifically in the mechanosensory neurons. In this case, we observed a strong, but partial rescue of the enhanced axon termination defects in *fsn-1; anc-1* double mutants (compare 20.5±1.9% for *fsn-1; anc-1*+P*mec-7*::ANC-1 with 37.6±3.2% for *fsn-1; anc-1*, Figure 4C). Transgenic expression of ANC-1 in the surrounding muscle cells using the *myosin 3* (*myo-3*) promoter did not rescue defects in *fsn-1; anc-1* double mutants (Figure 4C).

Our transgenic analysis supports several conclusions. First, the dominant negative and rescue experiments demonstrate that ANC-1 functions cell autonomously in the mechanosensory neurons to regulate axon termination. Second, our results with the dominant negative indicate that ANC-1 needs to be associated with the nuclear envelope via its C-terminal KASH domain in order to regulate axon termination. Finally, the lesion in *anc-1* causes the enhanced axon termination defects observed in *fsn-1; anc-1* double mutants.

### *bar-1* functions through *pop-1* to regulate axon termination in the mechanosensory neurons

**Figure 5:**
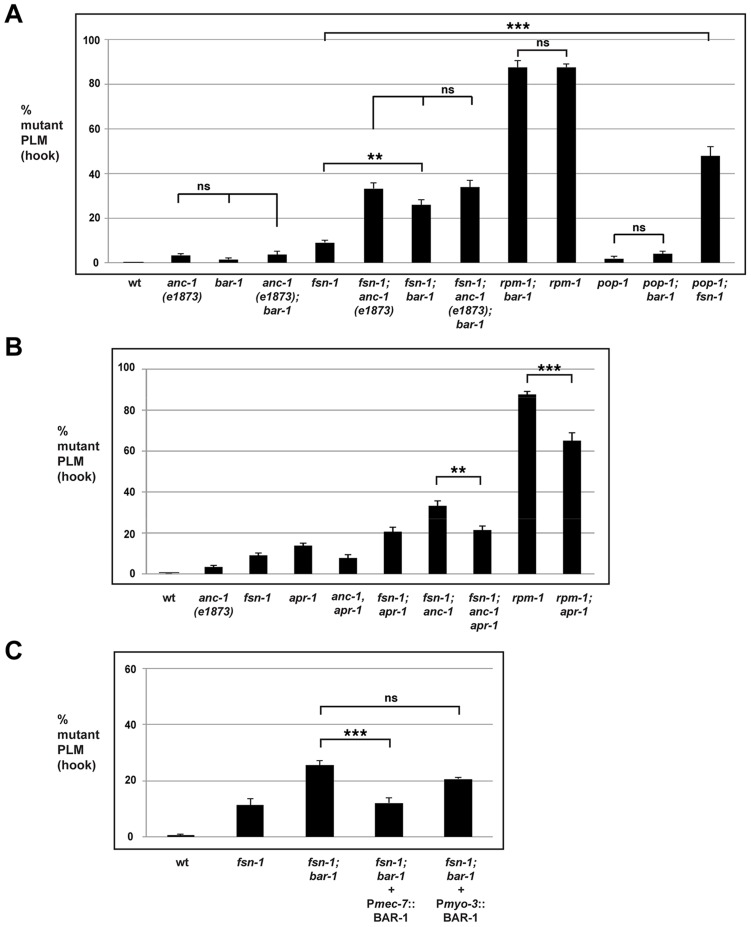
*bar-1* functions downstream of *anc-1* and *rpm-1* to regulate axon termination. Quantitation of axon termination defects (hook) in PLM neurons for the indicated genotypes using *muIs32*. (A) *bar-1* and *pop-1* analysis. (B) *apr-1* analysis. (C) A cell specific promoter (*Pmec*-7) was used to transgenically express BAR-1. Analysis was done on young adults grown at 23°C. Significance was determined using an unpaired Student's *t* test; error bars represent the standard error of the mean. **P<0.01, ***P<0.001, ns = not significant.

Our genetic analysis indicated a role for *bar-1* in synapse formation, and we next sought to determine if *bar-1* also functions in axon termination of the mechanosensory neurons, similar to *anc-1*. While axon termination defects were observed at very low penetrance in the PLM neurons of *bar-1* (lf) mutants, these defects did not reach statistical significance (Figure 5A). However, the penetrance of axon termination defects was enhanced in *bar-1; fsn-1* double mutants (25.8±2.5% hook) compared to *fsn-1* single mutants (9.1±1.1%, Figure 5A). This level of enhancement was similar to what we observed in *fsn-1; anc-1* double mutants, which suggested that *bar-1* might function in the same pathway as *anc-1* to regulate axon termination. To test this, we generated *anc-1; bar-1* double mutants and *fsn-1; anc-1; bar-1* triple mutants. As shown in Figure 5A, *anc-1; bar-1* double mutants did not show enhanced PLM axon termination defects. Similarly, *fsn-1; anc-1; bar-1* triple mutants were not enhanced compared to *fsn-1; bar-1* double mutants and *fsn-1; anc-1* double mutants (Figure 5A). Similar results were observed for *bar-1* regarding axon termination in the ALM neurons (Figure S3B). Thus, *bar-1* and *anc-1* regulate axon termination by functioning in the same genetic pathway.

Given that BAR-1 regulates the transcription factor POP-1, we also tested if *pop-1* functions in axon termination. Similar to *bar-1*, *pop-1* (lf) mutants had axon termination defects that occurred with very low penetrance, and *pop-1; fsn-1* double mutants were enhanced compared to single mutants (compare 47.7±4.3% for *pop-1; fsn-1* with 9.1±1.1% for *fsn-1*). Importantly, *pop-1; bar* double mutants were not enhanced consistent with *pop-1* and *bar-1* functioning in the same genetic pathway (Figure 5A). Consistent with the results from PLM neurons, ALM axon termination defects were enhanced in *pop-1; fsn-1* double mutants, but failed to show enhancement in *pop-1; bar-1* double mutants (Figure S3B). These results are consistent with *bar-1* functioning through *pop-1* to regulate axon termination.

To address if *bar-1* and *rpm-1* function in the same genetic pathway to regulate axon termination, we generated *rpm-1; bar-1* double mutants. As shown in Figure 5A, *rpm-1; bar-1* double mutants had similar penetrance of hook defects to *rpm-1* single mutants.

Given that ANC-1 functions cell autonomously in the mechanosensory neurons, we also wanted to determine if BAR-1 functions cell autonomously. To do so, we generated *fsn-1; bar-1* double mutants that transgenically express BAR-1 using a cell specific promoter. We performed our analysis on *fsn-1; bar-1* double mutants because the penetrance of axon termination defects were higher than in *bar-1* single mutants. When BAR-1 was expressed using a mechanosensory neuron specific promoter, P*mec-7*, the axon termination defects in *fsn-1; bar-1* double mutants were significantly reduced (compare 12.1±2.0% for *fsn-1; bar-1*+P*mec-7*::BAR-1 with 29.6±3.1% for *fsn-1; bar-1*, Figure 5C). In contrast, expression of BAR-1 with the *myo-3* promoter (expressed in body wall muscles surrounding the PLM axon) did not rescue defects in *fsn-1; bar-1* double mutants (Figure 5C).

Overall, these findings support several conclusions. First, *bar-1* regulates axon termination in the mechanosensory neurons by functioning in the same genetic pathway as *rpm-1*, *anc-1*, and *pop-1*. Second, *bar-1* functions in a parallel genetic pathway to *fsn-1*. Finally, *bar-1* regulates axon termination by functioning cell autonomously in the mechanosensory neurons.

### *bar-1* functions downstream of *anc-1* and *rpm-1* to regulate axon termination

Our genetic analysis indicated that *rpm-1*, *anc-1* and *bar-1* function in the same pathway. We next sought to determine whether *bar-1* functions up or downstream of *anc-1* and *rpm-1*. We chose to perform our epistasis analysis using *bar-1* for two reasons. First, the existing knowledge of how BAR-1 functions allowed the design and implementation of highly informative experiments. Second, a previous study in mammalian cells on Nesprin-2 and β-catenin (the orthologs of ANC-1 and BAR-1, respectively) suggested that *bar-1* might function downstream of *anc-1* [24]. Therefore, axon termination defects caused by loss of function in *rpm-1* and enhanced axon termination defects in *fsn-1; anc-1* double mutants might be due, in part, to decreased BAR-1 activity. If this model is correct, we anticipated that excess BAR-1 activity might suppress the axon termination defects caused by loss of function in *rpm-1*, and suppress the enhanced axon termination defects in *fsn-1; anc-1* double mutants. We chose to address this question using a genetic approach that utilized a loss of function mutation in *apc related 1 (apr-1)*, as GFP expressed by *muIs32* was greatly reduced (preventing proper visualization of mechanosensory neurons) when BAR-1 was transgenically overexpressed at high levels (data not shown).

APR-1 is the *C. elegans* ortholog of human Adenomatous Polyposis Coli (APC) [66]. In the vertebrate canonical Wnt signaling pathway, APC forms a complex with the scaffold protein Axin and Glycogen Synthase Kinase 3β (GSK3β) to phosphorylate β-catenin and target it for destruction [51]. APR-1 interacts with the functional axin ortholog Polyray 1 (PRY-1), and has been shown to negatively regulate the β-catenin BAR-1 during vulva development and neuroblast migration [67]. Thus, APR-1 can negatively regulate BAR-1 signaling, and *apr-1* mutants are likely to have increased levels of BAR-1 which is consistent with findings from other organisms on Wnt signaling and APC function.

Because axon termination defects in *anc-1* mutants occur with relatively low penetrance, we chose to analyze *fsn-1; anc-1* double mutants, which have enhanced penetrance of defects. As shown in Figure 5B, *fsn-1; anc-1; apr-1* triple mutants had significantly reduced penetrance of hook defects (21.4±1.9%) compared to *fsn-1; anc-1* double mutants (33.2±2.4%). Importantly, *fsn-1; apr-1* double mutants did not show reduced penetrance of defects (Figure 5B). These results show that increased BAR-1 activity suppresses *anc-1* (lf), which is consistent with *bar-1* functioning downstream of *anc-1*.

To test if *bar-1* functions downstream of *rpm-1* we took a similar genetic approach using *apr-1*. As shown in Figure 5B, *rpm-1; apr-1* double mutants showed suppressed penetrance of axon termination defects when compared to *rpm-1* single mutants (compare 65.1±3.9% hook for *rpm-1; apr-1* with 87.6±1.5% for *rpm-1*). These results demonstrate that *bar-1* is likely to function downstream of *rpm-1*.

### *unc-84* regulates axon termination

Our observation that transgenic overexpression of the ANC-1 KASH domain in *fsn-1* mutants results in enhanced axon termination defects suggested that ANC-1 needs to be at the nuclear envelope in order to regulate axon termination (Figure 4C). To further support this concept, we examined the role of UNC-84 in axon termination. UNC-84 is a conserved SUN domain protein that is localized to the inner nuclear membrane. UNC-84 binds to ANC-1, thereby tethering ANC-1 in the nuclear envelope and mediating formation of the LINC complex [6], [68]. Therefore, we hypothesized that if ANC-1 needs to be localized to the nuclear membrane to function in axon termination, then *unc-84* should regulate axon termination similar to *anc-1*.

**Figure 6:**
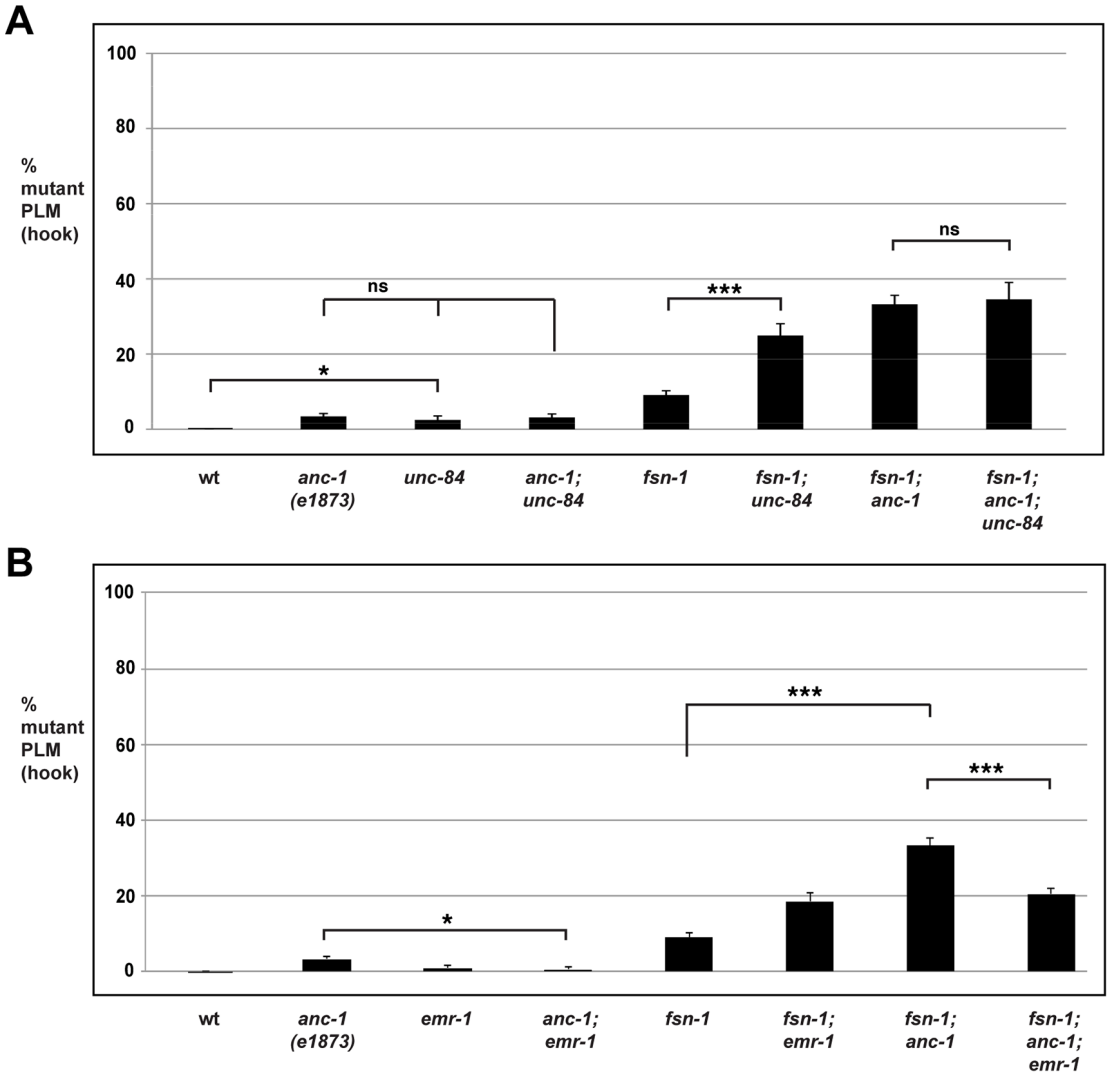
*anc-1* and *unc-84* function in the same genetic pathway to regulate axon termination. Quantitation of axon termination defects (hook) in PLM neurons for the indicated genotypes using *muIs32*. (A) *unc-84* mutant analysis. (B) *emr-1* mutant analysis. Analysis was done on young adults grown at 23°C. Significance was determined using an unpaired Student's *t* test; error bars represent the standard error of the mean. *P<0.05, ***P<0.001, ns = not significant.

In *unc-84* (lf) mutants we observed a very mild penetrance of hook defects, similar to *anc-1* (lf) animals (Figure 6A). *fsn-1; unc-84* double mutants had enhanced penetrance of defects compared to *fsn-1* single mutants (compare 24.9±3.1% hook for *fsn-1; unc-84* with 9.1±1.1% for *fsn-1*, Figure 6A). In contrast, *anc-1; unc-84* double mutants did not show a significant increase in penetrance compared to single mutants (Figure 6A). Likewise, the penetrance of defects in *fsn-1; anc-1; unc-84* triple mutants was not increased compared to *fsn-1; anc-1* and *fsn-1; unc-84* double mutants (Figure 6A). The *unc-84* allele we used, *e1410*, is a hypomorph that results in loss of function in the SUN domain of UNC-84, and has nuclear anchorage defects [68]. Previous studies have shown that the SUN domain of UNC-84 is required for recruitment of ANC-1 to the nuclear envelope [6]. Thus, *unc-84(e1410)* is predicted to lack nuclear localization of ANC-1. This is consistent with our genetic data showing that loss of function in *unc-84* does not enhance loss of function in *anc-1*. Thus, *unc-84* regulates axon termination by functioning in the same genetic pathway as *anc-1*, and in a parallel pathway to *fsn-1*. These findings are also consistent with ANC-1 regulating axon termination by functioning at the nuclear envelope.

### *anc-1* functions through *emr-1* to regulate axon termination

It was previously shown that Emerin binds to and facilitates the nuclear export of β-catenin thereby antagonizing β-catenin function [69]. Emerin also binds to Nesprin-1 and Nesprin-2 [70], [71]. These prior observations suggested that ANC-1 might regulate BAR-1 by functioning through Emerin homolog 1 (EMR-1) in *C. elegans*. To test this, we analyzed the genetic relationship between *anc-1* and *emr-1* using *gk119*, an allele that deletes the entire *emr-1* coding sequence [72]. Although PLM axon termination defects occurred with very low penetrance in *anc-1* single mutants, defects in *anc-1; emr-1* double mutants were significantly suppressed (compare 3.4±0.8% hook for *anc-1* with 0.6±0.6% for *anc-1; emr-1*, Figure 6B). Consistent with this result, we also observed that the enhanced penetrance of axon termination defects in *fsn-1; anc-1* double mutants was suppressed in *fsn-1; anc-1; emr-1* triple mutants (compare 33.2±2.2% hook for *fsn-1; anc-1* with 20.5±1.5% for *fsn-1; anc-1; emr-1*, Figure 6B). Notably, *fsn-1; emr-1* double mutants were mildly enhanced rather than being suppressed (Figure 6B). This result explains why *fsn-1; anc-1; emr-1* triple mutants were only suppressed to the level of defect present in *fsn-1; emr-1* double mutants. Our findings demonstrate that *anc-1* and *emr-1* function in the same genetic pathway, and that ANC-1 is a negative regulator of EMR-1.

### Wnt signaling regulates PLM and ALM axon termination

Our observation that RPM-1 and ANC-1 function through BAR-1, which mediates canonical Wnt signaling, prompted us to test which Wnt ligands regulate axon termination in the ALM and PLM mechanosensory neurons. To do so, we analyzed loss of function alleles in four Wnt ligands: *c. elegans wnt family 1 (cwn-1)*, *cwn-2*, *egg laying defective 20 (egl-20)*, and *lin-44*. The fifth Wnt, *more of ms 2 (mom-2)*, was not analyzed because loss of function is lethal.

With regard to the ALM neurons, loss of function in individual Wnt ligands did not result in significant defects in axon termination (Figure S3C). Analysis of double mutants with *fsn-1* showed that only *fsn-1; cwn-2* double mutants had enhanced axon termination defects (Figure S3C).

**Figure 7:**
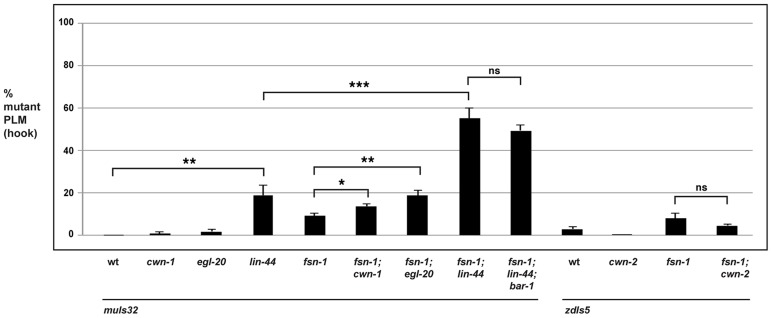
The Wnts *egl-20* and *lin-44* function through *bar-1* to regulate PLM axon termination. Quantitation of axon termination defects (hook) in PLM neurons for the indicated genotypes using *muIs32*. Note that *zdIs5* (P_mec-4_GFP) was used for *cwn-2* analysis because both *muIs32* and *cwn-2* are on chromosome II. Analysis was done on young adults grown at 23°C. Significance was determined using an unpaired Student's *t* test; error bars represent the standard error of the mean. *P<0.05, **P<0.01, ***P<0.001, ns = not significant.

In the PLM neurons, the situation was more complex. *cwn-1* and *cwn-2* single mutants did not show defects in PLM axon termination. While *fsn-1; cwn-2* double mutants failed to show enhancement, *fsn-1; cwn-1* double mutants showed a small but significant enhancement (compare 13.6±1.3% hook defects for *fsn-1; cwn-1* with 9.1±1.1% for *fsn-1*, Figure 7). In the case of *egl-20*, single mutants did not have a significant defect, but mild enhancer effects were observed in *fsn-1; egl-20* double mutants (compare 18.8±2.3% hook defects for *fsn-1; egl-20* with 9.1±1.1% for *fsn-1*, Figure 7). Consistent with previous work, we observed that axon polarization was abnormal in the PLM neurons of *lin-44* mutants (data not shown) [54], [73]. However, axon polarization defects were not completely penetrant in *lin-44* mutants, which allowed us to analyze axon termination in neurons with normal polarity. Using this approach, we observed that *lin-44* mutants had significant defects in PLM axon termination (compare 18.9±4.7% hook for *lin-44* with 0% defects for wild-type, Figure 7). Further, *fsn-1; lin-44* double mutants were significantly enhanced compared to *fsn-1* or *lin-44* single mutants (compare 55.1±4.8% hook for *fsn-1; lin-44* with 18.9±4.7% for *lin-44*, Figure 7). Consistent with LIN-44 functioning through BAR-1 to regulate axon termination, we observed no further enhancement of defects in *fsn-1; lin-44; bar-1* triple mutants (Figure 7). Notably the more posterior the location of expression for a Wnt ligand the stronger the phenotypes and/or enhancer effects observed, *e.g. lin-44* mutants had the strongest PLM axon termination defects and enhancer effects, and LIN-44 is the most posteriorly expressed Wnt [74].

Thus, because the ALM and the PLM neurons terminate axon extension in anatomically distinct locations, different Wnt ligands control this process for each type of neuron. Our results show that only *cwn-2* regulates ALM axon termination. In contrast, a combination of *cwn-1*, *egl-20* and *lin-44* regulate PLM axon termination with *lin-44* functioning most prominently.

## Discussion

**Figure 8:**
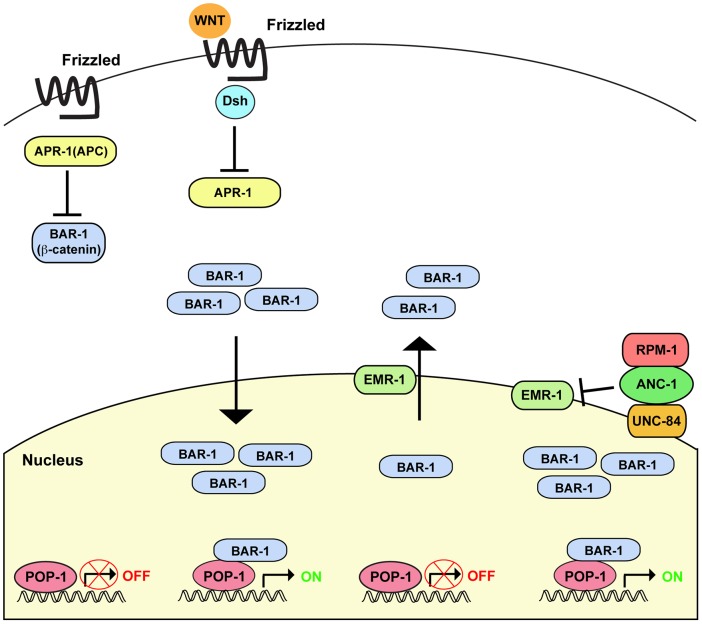
Summary of RPM-1 signaling through the ANC-1/BAR-1 pathway. Canonical Wnt signaling through Disheveled (Dis) and APR-1 (APC ortholog) regulates the β-catenin BAR-1. In the absence of Wnt, APR-1 is active and inhibits BAR-1. In the presence of Wnt, APR-1 is inhibited and BAR-1 activity is increased. Higher levels of BAR-1 lead to increased nuclear import and activation of the transcription factor POP-1 (TCF/LEF). We have shown that RPM-1 binds to ANC-1, and RPM-1 and ANC-1 function in the same pathway to positively regulate BAR-1 activity. RPM-1 and ANC-1 are likely to function in a protein complex at the nuclear envelope to regulate BAR-1 nuclear levels by inhibition of EMR-1. Canonical Wnt signaling is likely to act coordinately with RPM-1 and ANC-1 to regulate BAR-1 activity.

While the role of the Nesprins in nuclear positioning and movement is well established, mounting evidence has suggested additional roles for this protein family. High expression of Nesprin-1 in the adult brain, links to synaptic plasticity, and an association with neurological conditions suggest that the Nesprins may function in neuronal development. Here, we show that ANC-1 binds to the PHR protein RPM-1, a large signaling protein involved in numerous developmental events in neurons. Our genetic analysis indicates that ANC-1 functions cell autonomously to regulate axon termination in the mechanosensory neurons, and synapse formation in the GABAergic motor neurons. We also show that ANC-1 functions by positively regulating BAR-1, the β-catenin isoform that functions in canonical Wnt signaling (Figure 8). Our study reveals a novel role for ANC-1 in intracellular signaling and neuronal development. Importantly, we have also identified the ANC-1/BAR-1 pathway as a new mechanism by which RPM-1, and possibly other PHR proteins, regulate axon termination and synapse formation.

### ANC-1 mediates RPM-1 function

Our proteomic screen for RPM-1 binding proteins identified the nuclear anchorage protein ANC-1, which was confirmed using coIP. Consistent with these findings, our genetic analysis indicated that *anc-1* and *rpm-1* function in the same genetic pathway to regulate synapse formation in the GABAergic motor neurons, and axon termination in the mechanosensory neurons. Transgenic analysis indicated that *anc-1* functions cell autonomously in the mechanosensory neurons to regulate axon termination, similar to *rpm-1*. Our observation that defects in *anc-1; rpm-1* double mutants were not suppressed also suggests that RPM-1 positively regulates ANC-1.

While we were unable to provide definitive evidence that *anc-1* functions downstream of *rpm-1*, our analysis indicates that this is likely to be the case. We show that *bar-1* functions downstream of both *anc-1* and *rpm-1*, and that these three genes function in the same genetic pathway. Because similar enhancer effects are observed in *fsn-1; bar-1* and *fsn-1; anc-1* double mutants, and the penetrance of these defects is less than *rpm-1* (lf) mutants, it is probable that *anc-1*, like *bar-1*, functions downstream of *rpm-1*.

The PHR protein family is highly conserved with orthologs in Drosophila, zebrafish and mice [25]. To date, all of the proteins identified in our proteomic screen for RPM-1 binding proteins (including FSN-1, GLO-4, and RAE-1) are evolutionarily conserved [42], [43]. Therefore, RPM-1 and its downstream signaling pathways are likely to function through conserved mechanisms. ANC-1 is also conserved with orthologs in Drosophila (MSP-300) and mammals (Nesprin-1 and 2). Thus, the function of ANC-1 in axon termination and synapse formation during development is also likely to be conserved. This is supported by the observation that the branches of phrenic nerves are overgrown in Nesprin-1^−/−^ knockout mice, indicating possible axon termination defects [2]. Further addressing if the role of ANC-1 in axon and synapse development is evolutionarily conserved remains an important goal.

### ANC-1 functions through the β-catenin BAR-1

Our genetic results showed that the β-catenin *bar-1* functions in synapse formation in the GABAergic motor neurons, and axon termination in the mechanosensory neurons. Prior studies showed that BAR-1 regulates glutamate receptor trafficking, and axon extension in motor neurons [55], [75]. Our results show that BAR-1 plays a more expansive role in neuronal development than originally thought. We also provide significant insight into the mechanism of how BAR-1 is regulated by showing that a novel pathway containing RPM-1 and ANC-1 functions upstream of BAR-1. Our functional genetic and transgenic findings are consistent with a prior study on a keratinocyte cell line, which showed that Nesprin-2 regulates the nuclear localization of β-catenin [24]. Our results do not rule out the possibility that ANC-1 regulates the ubiquitination or phosphorylation of BAR-1. We used epifluorescent microscopy to investigate if BAR-1::GFP localization to the nucleus was altered in *anc-1* mutants, but found no obvious changes (data not shown). However, it is possible that detecting such changes may require more sensitive methods.

In the canonical Wnt signaling pathway, BAR-1 activates POP-1 (the *C. elegans* TCF/LEF transcription factor) [58]. Our observation that defects in axon termination and synapse formation were enhanced in both *fsn-1; pop-1* and *fsn-1; bar-1* double mutants, and that defects were not enhanced in *pop-1; bar-1* double mutants is consistent with *bar-1* and *pop-1* functioning in the same genetic pathway. Because *fsn-1; pop-1* double mutants displayed more penetrant defects than *fsn-1; bar-1* double mutants, it is possible that another β-catenin may also function through POP-1 to regulate axon termination. Humpback 2 (HMP-2) is an unlikely candidate as it functions in a cadherin-catenin complex and does not act through POP-1 [60]. Symmetrical Sister Cell and Gonad Defect 1 (SYS-1) and Worm Armadillo 1 (WRM-1) are plausible candidates as they regulate transcriptional activation and nuclear export of POP-1, respectively [76], [77]. In the case of synapse formation in the GABAergic motor neurons the situation is simpler. Phenotypes were similar in *fsn-1; pop-1* and *fsn-1; bar-1* double mutants suggesting that only BAR-1 is likely to function through POP-1 to regulate synapse formation in GABAergic motor neurons.

In vertebrates, β-catenin plays a broad and important role in neurons by regulating a range of processes including: neuronal differentiation [78], synaptic vesicle assembly [79], dendrite morphogenesis and plasticity [80], [81], neurite extension [82], and axon arborization and targeting in retinal ganglion cells (RGC) [83]. β-catenin also regulates the morphology of the NMJ and phrenic nerve growth [84]. Notably similar to the function of β-catenin, Phr1 regulates RGC axon arborization and targeting [31] and NMJ morphology [30], [33], and both Phr1 and Nesprin-1 regulate axon growth in phrenic nerves [2], [33]. While these studies hinted at a possible functional link between the PHR proteins, Nesprins and β-catenin, our finding that RPM-1, ANC-1 and BAR-1 function in the same pathway provides the first mechanistic explanation for these phenotypic relationships in mammals. Thus, the functional relationship between RPM-1, ANC-1 and BAR-1 is likely to be evolutionarily conserved. Further, our study outlines a signaling network that links two central and important regulators of neuronal development, the PHR proteins and β-catenin, through the function of ANC-1.

### ANC-1 regulates axon termination by functioning at the nuclear envelope

ANC-1 regulates nuclear anchorage by binding to the nuclear envelope via its C-terminal KASH domain, and binding to the actin cytoskeleton via its N-terminal calponin homology domains [6]. The SUN domain protein UNC-84 mediates binding of ANC-1 to the nuclear envelope. Several of our findings demonstrate that ANC-1 needs to be anchored to the nuclear envelope in order to regulate axon termination. First, we found that transgenic overexpression of dominant negative ANC-1, which acts by inhibiting recruitment of endogenous ANC-1 to the nuclear envelope [6], enhances the axon termination defects caused by *fsn-1* (lf). Second, loss of function in either *anc-1* or *unc-84* enhances the axon termination defects caused by *fsn-1* (lf). Third, our results demonstrate that *anc-1* and *unc-84* function in the same genetic pathway to regulate axon termination. Finally, axon termination defects in *anc-1* single mutants and *fsn-1; anc-1* double mutants are suppressed by loss of function in *emr-1*, a nuclear envelope associated protein. Collectively, these findings support the conclusion that ANC-1 regulates axon termination by functioning at the nuclear envelope.

Previous studies showed that RPM-1 is localized to the perisynaptic zone of presynaptic terminals [32], [85]. However, a transgenically expressed fusion protein of RPM-1 and GFP that rescues *rpm-1* (lf) phenotypes is also found at low levels in the cell body and excluded from the nucleus of the mechanosensory neurons (Opperman and Grill, *in press*) and the motor neurons (Figure S4) [32]. In addition, RPM-1 binds to RAE-1, a protein that localizes to the nuclear envelope as well as presynaptic terminals [43]. These prior observations combined with our findings here that ANC-1 binds to RPM-1, that both genes function in the same pathway, and that ANC-1 regulates axon termination by functioning at the nucleus suggest that RPM-1 may play a novel signaling function in the neuronal cell body, possibly at the nuclear envelope. This possibility is further supported by immunohistochemistry in mammals, which has shown that the rat ortholog of RPM-1 (called MYCBP2 or Pam) is present in the cell bodies of neurons in the spinal cord and throughout the brain [86], [87].

### RPM-1/ANC-1 signaling and Wnt function

An emerging question is whether the PHR proteins, or the signaling pathways they control, are regulated or integrated with signals originating from outside the cell. Extracellular guidance cues, adhesion molecules and morphogens, such as Wnts, play roles in both axon guidance and synapse formation [88]. A previous study in zebrafish noted that loss of function in Phr1 and Wnt4a/Ryk causes abnormal axon stopping at the medial habenula hinting at a possible link between PHR protein function and Wnt signaling [89]. We now provide genetic evidence linking the PHR protein RPM-1 to the canonical β-catenin BAR-1 and Wnt signaling. Specifically, we show that several Wnt ligands regulate axon termination by functioning coordinately with FSN-1, a key component of RPM-1 signal transduction. In the anterior of the animal, we found that only CWN-2 regulates ALM axon termination. Our finding is consistent with previous studies showing that CWN-2 regulates axon polarization in ALM neurons, and anterior axon guidance in neurons of the nerve ring [54], [90]. Further, CWN-2 regulation of ALM axon termination is consistent with CWN-2 being the most anteriorly expressed Wnt ligand [74]. With regard to the PLM neurons in the posterior of the animal, three Wnts are involved in decreasing order of importance: LIN-44, EGL-20, and CWN-1. These findings are consistent with several previous observations. First, this same combination of Wnt ligands was shown to regulate axon polarization in the PLM neurons [54], [73]. Second, the hermaphrodite specific motor neuron (HSN) cell bodies are in roughly the same relative anterior-posterior location as the sites of PLM axon termination, and the same three Wnt ligands we have found that control PLM termination also regulate HSN migration [53].

Our genetic analysis indicates that RPM-1 and ANC-1 positively regulate BAR-1. In mammals, Nesprin-2 binds to a complex of α- and β-catenin [24]. Thus, RPM-1 may mediate formation of an ANC-1/BAR-1 complex that regulates nuclear levels of BAR-1 (Figure 8). *In vitro* biochemical and tissue culture experiments have shown that Nesprin-1 and Nesprin-2 bind to the nuclear membrane protein Emerin, and Emerin regulates nuclear export of β-catenin [69], [71]. We have found that loss of function in *emr-1* suppressed *anc-1* (lf) in the context of axon termination, which is consistent with ANC-1 inhibiting EMR-1. Thus, excess EMR-1 activity in *anc-1* mutants presumably results in increased nuclear export of BAR-1, thereby mimicking loss of function in *bar-1*. Taken collectively, prior findings and our results here are consistent with a model in which canonical Wnt signaling controls BAR-1 protein levels and nuclear import, while RPM-1 and ANC-1 function in a linear pathway to inhibit EMR-1 and nuclear export of BAR-1 (Figure 8).

## Materials and Methods

### Genetics

The N2 isolate of *C. elegans* was propagated using standard procedures. Alleles used in this study included; *anc-1(e1873)*, *anc-1(e1753)*, *apr-1(ok2970)*, *pop-1(q645)*, *fsn-1(gk429)*, *glo-4(ok623)*, *rpm-1(ju44)*, *bar-1(ga80)*, *unc-84(e1410)*, *emr-1(gk119)*, *cwn-1(ok546)*, *cwn-2(ok895)*, *lin-44(n1792)*, and *egl-20(hu120)*. The strain MH1870, which contains the transgene *kuIs54*, and *anc-1(e1873)* were kind gifts from Dr. Daniel Starr. All mutants were constructed using standard procedures, and were confirmed using PCR or by the associated visible phenotypes. Heterozygous analysis with *rpm-1* was done by linking *rpm-1(ju44)* with *dpy-11(e224)*, and using *unc-42(e270)* as a balancer. Other homozygous alleles were isolated as necessary and non-dpy non-unc (*rpm-1 dpy-11/unc-42*) animals were scored. The transgenic strains used in this study were: *muIs32* [P*_mec-7_*GFP], *juIs1* [P_unc-25_SNB-1::GFP], and *kuIs54* [P_sur-5_SUR-5::GFP].

### Transgenics

Transgenic animals were generated using standard microinjection procedures. Transgenes were constructed by injection of plasmid DNA or DNA generated by PCR with plasmid encoding P_ttx-3_RFP (50 ng/µL) and pBluescript (50 ng/µL). Dominant negative ANC-1 was cloned as a genomic fragment (bp 36523–37778). For *rgef-1* promoter lines, the plasmid pBG-GY360 was amplified by PCR and injected at 10 ng/µL. For the *mec-3* promoter lines, the plasmid pBG-GY370 was amplified by PCR and injected at 5 ng/µL. The *anc-1* mini-gene was constructed by ligating together 4 fragments: a cDNA fragment from 1–4154 bp using an engineered ApaI site and BamHI, a BamHI to NotI genomic fragment (14,808–24,000), a NotI to KpnI genomic fragment (24,001–36,849), and a KpnI to 3′ UTR fragment containing an engineered SacII site (36,850–38,921). For *anc-1* rescue analysis, a plasmid encoding P*_mec-7_*ANC-1 (mini-gene, pBG161) was injected at 10–40 ng/µL, and a plasmid encoding P*_myo-3_*ANC-1 (mini-gene, pBG-183) was injected at 20 ng/µL. For *bar-1* rescue analysis, a plasmid encoding P*_mec-7_*BAR-1 (genomic clone, pBG-GY318) was injected at 1 ng/µL.

### Analysis of axon termination and synapse formation

Analysis was carried out on live animals at 40× magnification using a Nikon epifluorescent microscope and a Q-imaging camera. Animals were anesthetized using 1% (v/v) 1-phenoxy-2-propanol in M9 buffer. Synapse formation defects were quantified by collecting images of *juIs1* (P_unc-25_SNB-1::GFP) and manually scoring puncta numbers in Adobe Photoshop. Dorsal cord lengths were determined in µmeters using Q-imaging software. For each genotype 20 or more worms were analyzed from at least 3 independent experiments. Both *bar-1* and *pop-1* mutants displayed stereotyped, reproducible gaps in their dorsal cords (data not shown). Care was taken to avoid collecting images at these locations. Axon termination defects were visualized using *muIs32* (P_mec-7_GFP) and manually scored. For all genetic analysis on axon termination, averages are shown for data collected from 5–8 independent counts of 20–30 PLM neurons from young adult worms. For transgenic analysis, data shown is an average of 4 or more transgenic lines for each genotype.

### Biochemistry

Proteomic analysis of RPM-1 binding proteins, including ANC-1, was described previously [42]. For biochemical analysis of RPM-1 binding to ANC-1, worms were grown in liquid culture, harvested by centrifugation, frozen in liquid N2, ground with a mortar and pestle under liquid N2, and extracted using 0.1% NP-40 lysis buffer as described previously [42]. CoIPs were performed from 40 mg of total protein extract. RPM-1::GFP was precipitated using a mouse monoclonal antibody (3E6, MP Biomedical) and protein G agarose beads (Roche). For immunoblotting, precipitates were run on an SDS-PAGE gel (3–8% Tris Acetate, Invitrogen), and proteins were wet transferred to PVDF membrane in Tris acetate transfer buffer (30 volts for 24–30 hours). Blots were blocked with part-skim milk in TBST, and probed with an anti-GFP antibody (mouse monoclonal, Roche) or purified anti-ANC-1 polyclonal antibodies that were used previously [6]. Primary antibodies were detected with secondary antibodies coupled to HRP, Supersignal FemtoWest enhanced chemiluminescent reagent (Pierce), and autoradiography.

## Supporting Information

Figure S1 Summary of proteomic results for ANC-1. Shown is the protein sequence of ANC-1. Highlighted are the calponin homology domains (red), the repeat regions (shaded alternating beige and grey), the KASH domain (yellow), and the sequences corresponding to peptides identified by mass spectrometry (blue and green). Note that while only 10 unique peptides were identified, because ANC-1 contains large amounts of repeated sequence, these peptides are highlighted in multiple locations.
(PDF)

Figure S2 Further examples of ANC-1 coIP with RPM-1. Shown are two independent examples of coIPs from transgenic worm lysates showing that endogenous ANC-1 binds to RPM-1::GFP.
(PDF)

Figure S3 *anc-1*, *bar-1* and the Wnt *cwn-2* regulate axon termination in the ALM mechanosensory neurons. ALM axon termination defects were quantitated for the indicated genotypes using *muIs32* [P_mec7_GFP]. (A) *anc-1* mutant analysis (B) *bar-1* mutant analysis. (C) *cwn-2* mutant analysis. Note that *zdIs5* (P_mec-4_GFP) was used for *cwn-2* analysis because both *muIs32* and *cwn-2* are on chromosome II. Analysis was done on young adults grown at 23°C. Significance was determined using an unpaired Student's *t* test; error bars represent the standard error of the mean. *P<0.05, **P<0.01, ***P<0.001, ns = not significant.
(TIF)

Figure S4 RPM-1::GFP localizes at low levels in neuronal cell bodies, but is excluded from the nucleus. Epifluorescent microscopy was used to visualize transgenic animals, *juIs58*, in which RPM-1::GFP was expressed using the native *rpm-1* promoter. In the ventral cord motor neurons, RPM-1::GFP was concentrated at the presynaptic terminals (arrowheads). RPM-1::GFP was also localized at low levels in the cell bodies of motor neurons, where it was excluded from the nucleus (arrows).
(PDF)
