# Supplementary material for: The Nesprin Family Member ANC-1 Regulates Synapse Formation and Axon Termination by Functioning in a Pathway with RPM-1 and β-Catenin
Source: PLoS Genet. 2014 Jul 10;10(7):e1004481. doi: 10.1371/journal.pgen.1004481 (PMC4091705; doi:10.1371/journal.pgen.1004481)
Supplement: Figure S1 — Summary of proteomic results for ANC-1. Shown is the protein sequence of ANC-1. Highlighted are the calponin homology domains (red), the repeat regions (shaded alternating beige and grey), the KASH domain (yellow), and the sequences corresponding to peptides identified by mass spectrometry (blue and green). Note that while only 10 unique peptides were identified, because ANC-1 contains large amounts of repeated sequence, these peptides are highlighted in multiple locations. (PDF) [file pgen.1004481.s001.pdf]

Tulgren et al — Supplementary Figure 1

ANC-1

MSSSPPARPCCVCFRFRPHEDEKAQKNTFTRWINFHLEEHSSSGRIEDLFEDIRDGVLLCHLIEVLTGEALAVHKGRVSKRVHHIANLTTALTVLRRRGLELINNNAADIADGNPRIVLGLIW
QIILHFQIETNMILLREWGWAATGTEEPTSSAQPEVVVTAPSPTPSSKKSHSKVSSLSGSKTSLASGEKAPSSPLRQRIASFLTPTKKAPKLTAQPVKQSVEQVFLRWINAEIGDLVGGRRVE
NMDKQWRDGILFCALVSRWRPDVISMREVTNANPRDNLELAFNLAHQHLGVRRLLAVEDMMIEKPDKRSVITYVSQFVRMFGERSPMQGREQHEIFLAWLEATYLLCTRHELNSQECSR
IRREFIEHRPLFNTIIVTKVNYDVEELVEIEKKWDCIRETLEKYARRSERDLPEPFASIATWIAGAEHILSRPLDLDATDAKKTVTMLQKLISEHQKYMEDLPKRQEDFEEAVKHGGLGGRPVA
PEFSEPLRARFAQIEEENEPRISTLRILTTHYILLQYLQHIDEKIILWRTADSVTLLLRWIKEYTQLNAENPQAKCASYINKITLTMANDQNSKLDKEAILNTSNEKTAETLKRFESLWIELKLLKVE
WVEWETHVSQLEEIIEERRRNGVPPTPEDEQALAVITAGADQLAPKLGASARLSNNQRLDVLTHSFKKLTKTTIKIGGRLVVELEPSTSEQASKISYSWQASDELLKVEMQLRDRIQRDADS
SDREQLEYRAETFRNIRDKLIELERLNEIYDNHSRDQVDTPNRNLIRHDMGTIIYGLQAGQYANFVDLSCYAFVYDEYNQVPVNLTENVLSEDYVREIVNRKKAILTRRENNEDEIRESIRDM
EECDRIIEGWQSTEIEELRAEWNIKLSEFESWHEMMQQVEVLSQTIQTRLDVTVIQTIWILKERSYDIKHSELGGTLRESLEQLATTSETSVNRHLQNLELSNEQDCPDAIEFLEEVGRESVS
KLSAAVDDRYIYTLHVLRTKMELFRRLQNFCDAVKILRSQNTKWNGIKISQIDQVQSEIDTLIVRLDEEWTQDANQLRAELASIHGSFFQLEFDRLNEKLNMLIHDKDKLRELMVHRRHYLTA
ANELITDSKTDLAQRATSSDHPDDILRATDEVTKALDIKGEELRRLGELAEMNITDLVVVALILSFRRRQLGSEGEPEVEELRRALREIIARPITEPRDVSPDAIVADILRMKDEKKRDEKTIDEI
QATTLTDEQRASFAPLIEDYRRRADRHRIVFEHLVMIYLDWLSRQFDELEDEIGMTIQTSRADDLRRMNSTEWNKWKTDLANIERQVGPDTKKALSAELADLHRKRDSMEARINKYLTHSA
KINAKLTQFEKWLNAIEEDIEQTERQFEDPERSYRFGSLHEVALAKQRLVAKLERLNVANKEEVLRLCERYHTIMHKLTPFQTAVGLPLHVSTNLDRNGPFQSQISVSSIASSELERPESVMS
LTSSIGVIPADVAELSPFEAKINKLLQKLHIIEDSYLKGPKPIDTVREDVKQLEKYRNRGAEILQQLSTSNIEDAEKEGLKHRFVLMLNNYDDILRSIENEIRDDNELTAKNQEILAELSNAEQTL
QNSPLEDLDISAELDRLQMQLDLVKVMCNKPRKYVECELIDSSREGSPQERRRRKKVMVMVSNTITTIIHVVEERLEASDISRNPDVQQKLVAVKENLRELDTTTVTPHPPSIMSPLGTEN
RNDLEEVKRLAAEIDRAIDTASSMYEDAPTDEDALKSAIHLLDDQKVTLNHLHVVLEGIPEKNEQDRTDAIDIASSVGEKLGNLKSAVEEVYEEVLASTNPVKEDQPLQHIQEVQTTPAEQSN
WDTDEFSRQPPVLSDERIELK**TDPSAIDK**FEVRSDEDPVPKIIDLFGQLQTAVNEASPLACEGTDDVDALQVASDKLTKQDRTIRKIHAILDTIDDPVQKPAIIESLDKIKDQINNARDNINRQID
NLNYNQTPVVVPKESTKTPLNEIEDAVRQASTVVSDELCNTEKLLSARQTLANVKPQVDSVEANVWSNPETIETVTPILEEYTTLVDNIEKKLANEIEVPTNDPSRQDLVQQLQDVILECEEV
VVNCDNIEKLEESKLKLEKARPLLDQIGDNVEKLSREQSPDTSDAIDALSNVHQQYNATIMSIDDKIDELKNPEEDTSAADQLISELHVISEMPAVTIDLSMLNAIEEGLSTLPAHQAENVQAKI
DELRQKKEVADQTEQILSDLNAFGDMPAITLDLDLLKSVEDGIAVLPVEDSERIKAKIVDLRKKKEDADQAEALLQELSVISDMPISTLDLNMLQGIEDNLNSLPAEESDEIREKLNELRRRKQ
ESDQAEALLQELSVISDMPISTLDLNMLQGVEDNLNSLPTEEADKIREKINDLRRRKQESDLAEALLQELSVIYDMPTSTIDLNMLQGIEDNLNSLPAEESDKIREKINDLRRRKQESDLAEAL
LQELSVIYDMPTSTIDLNMLQGIEDNLNSLPAEESDKIREKINDLRRRKQESDQAEALLQELSVVSDMPASTIDINMLQSIEDGLSTLLSEDRSKIQQAIDSLRKKKSDSDLAQHALEALSVQS
KLPSVSINLEELKKLEETLSTVPVEDSKVIRDKIAELKTEKALADHAENYLVELKKIEDMPISAVGSDVLATIEDQILQMPVQYQPSVKETLDKLKQAKEEDDKLAGVYDELEKIAKLPARDYDN
KLLAKIDEKLNSLPKDQIAETHRKVEDIKVTKADIVAQIDVLDKLPAKDIDEHLLNSIEEKLPTIPSDSSDQLQIAIGKLRDRKQANIDEGKKILNELAEIQKMPADSLNEHALNLLATESDKFGSEI
SDKIMQEIDVLREKQNNHEVARLNAESVLQQLDKISEEPHLSLTEERLAPFLQNIDTVPACFVDKIRNKINEVQKLHDEAVQDEKDELKEKLVAKVQNIGKTSIDDVNVSDFEEIEREINGSLE
AFEAEPLLAKIQELREAKRVGDEARSAAHDQIVALEKEAEDVTAKESAKKKKKDKKKSPQEMIDELSAKVVEAKALIPKIEEAAKNENLPADDKPKAEQLVSNLEAFVKDVETQVSEKQDEL
DKLNNANDAIKRLGDALDDAEKTVVPSSVPALSEFKDRIAPHLATLVEAVNDVPASVEPSAVALRDRAAKFVSDLEKNIQKTGDDEKRADELKNDVGNAVKNVEDVVSKYQNQPQPLDVAK
DDANKLK**ATVEQLTKLAESSDKIDPQVAKD**IKDSKTKA**KELLQALEKA**IPQEDAIRREQAEINDRLNNLEKELTKVDEFKPEDALPIVDQLAANTNTLKTATDSNNEKAVAPSSLISHDDLVV
GLPEKVFQLQHAIDDKKQALN**KAAAVNEIAPKL**QLVSQQLQSVPQEVPASLDEQKQLLEDVENQKHNLENLLANLPENDPTADELRQKSQWDSLRLKDLLKQLGSAVGDKLAALAAFNAA
RKNAEDALLDITREDGGDDNKSPDELIDDLAKKEETVAKLLDTVSGVKPDELDDKERAEYNDLLA**RLATAADVLKN**KRAELEQAVKAKADEKSLHDSVDRIVSRLVPLVRESDELRHNAEA
VPTQYAPKAEELKKEVEAAKAVIANAPSSDAHVQQLEQAVATAETLIPDLEERAR**LWNEFLAAR**NDIDALIEQLQQPLDAVLAQPKRSAEEAAQDVENLRNNSQQLSDLDNKIANLQRISEL
LDPLESAYADV**RFFDVDAEQTRH**QYDDVLNDVAAELEDETLLKQSASQVANEIDDISKMIDSTDPER**SILDTIAK**SDIPALKAQINRIKDRIVNADASRKHVTTDP**KIAEDLDNKL**AKLQTELD
DAIKTSDEHDKEQLILSLKLNISQFEQIPLDQLKSDDLKTAEKEITNSLKPEEAEPLLAKIQELREAKRVGDEARSAAHDQIVALEKEAEDVTAKESAKKKKKDKKKSPQEMIDELSAKVVEAK
ALIPKIEEAAKNENLPADDKPKAEQLVSNLEAFVKDVETQVSEKQDELDKLNNANDAIKRLGDALDDAEKTVVPSSVPALSEFKDRIAPHLATLVEAVNDVPASVEPSAVALRDRAAKFVSDL
EKNIQKTGDDEKRADELKNDDGNAVKNVEDVVSKYQNQPQPLDVAKDDANKLK**ATVEQLTKLAESSDKIDPQVAKD**IKDSKTKA**KELLQALEKA**IPQEDAIRREQAEINDRLNNLEKELTK
VDEFKPEDALPIVDQLAANTNTLKTATDSNNEKAVAPSSLISHDDLVVGLPEKVFQLQHAIDDKKQALN**KAAAVNEIAPKL**QLVSQQLQSVPQEVPASLDEQKQLLEDVENQKHNLENLLAN
LPENDPTADELRQKSQWDSLRLKDLLKQLGSAVGDKLAALAAFNAARKNAEDALLDITREDGGDDNKSPDELIDDRGRSTGSAVGDKLAALAAFNAARKNAEDALLDITREDGGDDNKSP
DELIDDLAKKEETVAKLLDTVSGVKPDELDDKERAEYNDLLA**RLATAADVLKN**KRAELEQAVKAKADEKSLHDSVDRIVSRLVPLVRESDELRHNAEAVPTQYAPKAEELKKEVEAAKAVIA
NAPSSDAHVQQLEQAVATAETLIPDLEERAR**LWNEFLAAR**NDIDALIEQLQQPLDAVLAQPKRSAEEAAQDVENLRNNSQQLSDLDNKIANLQRISELLDPLESAYADV**RFFDVDAEQTRH**
QYDDVLNDVAAELEDETLLKQSASQVANEIDDISKMIDSTDPER**SILDTIAK**SDIPALKAQINRIKDRIVNADASRKHVTTDP**KIAEDLDNKL**AKLQTELDDAIKTSDEHDKEQLILSLKLNISQF
EQIPLDQLKSDDLKTAEKEITNSLKPEEAEPLLAKIQELREAKRVGDEARSAAHDQIVALEKEAEDVTAKESAKKKKKDKKKSPQEMIDELSAKVVEAKALIPKIEEAAKNENLPADDKPKAEQ
LVSNLEAFVKDVETQVSEKQDELDKLNNANDAIKRLGDALDDAEKTVVPSSVPALSEFKDRIAPHLATLVEAVNDVPASVEPSAVALRDRAAKFVSDLEKNIQKTGDDEKRADELKNDVGNA
VKNVEDVVSKYQNQPQPLDVAKDDANKLK**ATVEQLTKLAESSDKIDPQVAKD**IKDSKTKA**KELLQALEKA**IPQEDAIRREQAEINDRLNNLEKELTKVDEFKPEDALPIVDQLAANTNTLKT
ATDSNNEKAVAPSSLISHDDLVVGLPEKVFQLQHAIDDKKQALN**KAAAVNEIAPKL**QLVSQQLQSVPQEVPASLDEQKQLLEDVENQKHNLENLLANLPENDPTADELRQKSQWDSLRLK
DLLKQLGSAVGDKLAALAAFNAARKNAEDALLDITREDGGDDNKSPDELIDDLAKKEETVAKLLDTVSGVKPDELDDKERAEYNDLLA**RLATAADVLKN**KRAELEQAVKAKADEKSLHDSV
DRIVSRLVPLVRESDELRHNAEAVPTQYAPKAEELKKEVEAAKAVIANAPSSDAHVQQLEQAVATAETLIPDLEERAR**LWNEFLAAR**NDIDALIEQLQQPLDAVLAQPKRSAEEAAQDVENL
RNNSQQLSDLDNKIANLQRISELLDPLESAYADV**RFFDVDAEQTRH**QYDDVLNDVAAELEDETLLKQSASQVANEIDDISKMIDSTDPER**SILDTIAK**SDIPALKAQINRIKDRIVNADASRKH
VTTDP**KIAEDLDNKL**AKLQTELDDAIKTSDEHDKEQLILSLKLNISQFEQIPLDQLKSDDLKTAEKEITNSLKPEEAEPLLAKIQELREAKRVGDEARSAAHDQIVALEKEAEDVTAKESAKKKK
KDKKKSPQEMIDELSAKVVEAKALIPKIEEAAKNENLPADDKPKAEQLVSNLEAFVKDVETQVSEKQDELDKLNNANDAIKRLGDALDDAEKTVVPSSVPALSEFKDRIAPHLATLVEAVNDV
PASVEPSAVALRDRAAKFVSDLEKNIQKTGDDEKRADELKNDVGNAVKNVEDVVSKYQNQPQPLDVAKDDANKLK**ATVEQLTKLAESSDKIDPQVAKD**IKDSKTKA**KELLQALEKA**IPQE
DAIRREQAEINDRLNNLEKELTKVDEFKPEDALPIVDQLAANTNTLKTATDSNNEKAVAPSSLISHDDLVVGLPEKVFQLQHAIDDKKQALN**KAAAVNEIAPKL**QLVSQQLQSVPQEVPASLD
EQKQLLEDVENQKHNLENLLANLPENDPTADELRQKSQWDSLRLKDLLKQLGSAVGDKLAALAAFNAARKNAEDALLDITREDGGDDNKSPDELIDDLAKKEETVAKLLDTVSGVKPDEL
DDKERAEYNDLLA**RLATAADVLKN**KRAELEQAVKAKADEKSLHDSVDRIVSRLVPLVRESDELRHNAEAVPTQYAPKAEELKKEVEAAKAVIANAPSSDAHVQQLEQAVATAETLIPDLEER
AR**LWNEFLAAR**NDIDALIEQLQQPLDAVLAQPKRSAEEAAQDVENLRNNSQQLSDLDNKIANLQRISELLDPLESAYADV**RFFDVDAEQTRH**QYDDVLNDVAAELEDETLLKQSASQVANE
IDDISKMIDSTDPER**SILDTIAK**SDIPALKAQINRIKDRIVNADASRKHVTTDP**KIAEDLDNKL**AKLQTELDDAIKTSDEHDKEQLILSLKLNISQFEQIPLDQLKSDDLKTAEKEITNSLKPEEAE
PLLAKIQELREAKRVGDEARSAAHDQIVALEKEAEDVTAKESAKKKKKDKKKSPQEMIDELSAKVVEAKALIPKIEEAAKNENLPADDKPKAEQLVSNLEAFVKDVETQVSEKQDELDKLNN
ANDAIKRLGDALDDAEKTVVPSSVPALSEFKDRIAPHLATLVEAVNDVPASVEPSAVALRDRAAKFVSDLEKNIQKTGDDEKRADELKNDVGNAVKNVEDVVSKYQNQPQPLDVAKDDANK
LK**ATVEQLTKLAESSDKIDPQVAKD**IKDSKTKA**KELLQALEKA**IPQEDAIRREQAEINDRLNNLEKELTKVDEFKPEDALPIVDQLAANTNTLKTATDSNNEKAVAPSSLISHDDLVVGLPEKV
FQLQHAIDDKKQALN**KAAAVNEIAPKL**QLVSQQLQSVPQEVPASLDEQKQLLEDVENQKHNLENLLANLPENDPTADELRQKSQWDSLRLKDLLKQLGSAVGDKLAALAAFNAARKNAE
DALLDITREDGGDDNKSPDELIDDLAKKEETVAKLLDTVSGVKPDELDDKERAEYNDLLA**RLATAADVLKN**KRAELEQAVKAKADEKSLHDSVDRIVSRLVPLVRESDELRHNAEAVPTQY
APKAEELKKEVEAAKAVIANAPSSDAHVQQLEQAVATAETLIPDLEERAR**LWNEFLAAR**NDIDALIEQLQQPLDAVLAQPKRSAEEAAQDVENLRNNSQQLSDLDNKIANLQRISELLDPLE
SAYADV**RFFDVDAEQTRH**QYDDVLNDVAAELEDETLLKQSASQVANEIDDISKMIDSTDPER**SILDTIAK**SDIPALKAQINRIKDRIVNADASRKHVTTDP**KIAEDLDNKL**AKLQTELDDAIKT
SDEHDKEQLILSLKLNISQFEQIPLDQLKSDDLKTAEKEITNSLKPEEAEPLLAKIQELREAKRVGDEARSAAHDQIVALEKEAEDVTAKESAKKKKKDKKKSPQEMIDELSAKVVEAKALIPKI
EEAAKNENLPADDKPKAEQLVSNLEAFVKDVETQVSEKQDELDKLNNANDAIKRLGDALDDAEKTVVPSSVPALSEFKDRIAPHLATLVEAVNDVPASVEPSAVALRDRAAKFVSDLEKNIQ
KTGDDEKRADELKNDVGNAVKNVEDVVSKYQNQPQPLDVAKDDANKLK**ATVEQLTKLAESSDKIDPQVAKD**IKDSKTKA**KELLQALEKA**IPQEDAIRREQAEINDRLNKLEKELTKVDEFK
PEDALPIVDQLAANTNTLKTATDSNNEKAVAPSSLISHDDLVVGLPEKVFQLQHAIDDKKQALN**KAAAVNEIAPKL**QLVSQQLQSVPQEVPASLDEQKQLLEDVENQKHNLENLLANLPEND
PTADELRQKSQWDSLRLKDLLKQLGSAVGEKLAALAAFNAARKNAEDALLDITREDGGDDNKSPDELIDDLAKKEETVAKLLDTVSGVKPDELDDKERAEYNDLLA**RLATAADVLKN**KRA
ELEQAVKAKADEKSLHDSVDRIVSRLVPLVRESDELRHNAEAVPTQYAPKAEELKKEVEAAKAVIANAPSSDAHVQQLEQAVATAETLIPDLEERASIWERFVKAKDDLYDYLEKLENNVSD
VLNRPRLPVSQAQQRFNKLKEQSYLLDRIRDLKIDFDDLGEALLPLTVAEDELRFMHVHVESIERQYEDTMDKLNAEITAEVELLRTLDILSNELSQCKEDINNPSVDVDELSRATMLNDAIA
HLENQKVVVARSEKDRKFVESSTSIDLDQLLAEAKRLLKEIEPRLQLAQPDHDNEDDEDEEKGSDEKPYDVRAAAEVLSALYPDEHPHNVLRNIGFEELPSDSESRSEFDSLDSRSDGLLS
PIPDDSTLSEEQLRRQ**RSRWRRVLRTALPLQALLVLLMGAACLVPHCDDEYCCQLLNNFAKSFDPSLEFVNGPPPF**
