# Supplementary figures and images for: The Nesprin Family Member ANC-1 Regulates Synapse Formation and Axon Termination by Functioning in a Pathway with RPM-1 and β-Catenin
Source: PLoS Genet. 2014 Jul 10;10(7):e1004481. doi: 10.1371/journal.pgen.1004481 (PMC4091705; doi:10.1371/journal.pgen.1004481)

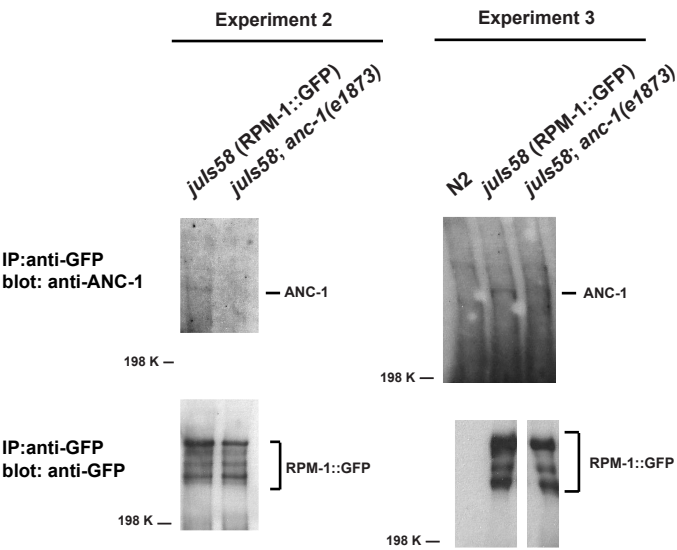

Supplement: Figure S2 — Further examples of ANC-1 coIP with RPM-1. Shown are two independent examples of coIPs from transgenic worm lysates showing that endogenous ANC-1 binds to RPM-1::GFP. (PDF) [file pgen.1004481.s002.pdf]

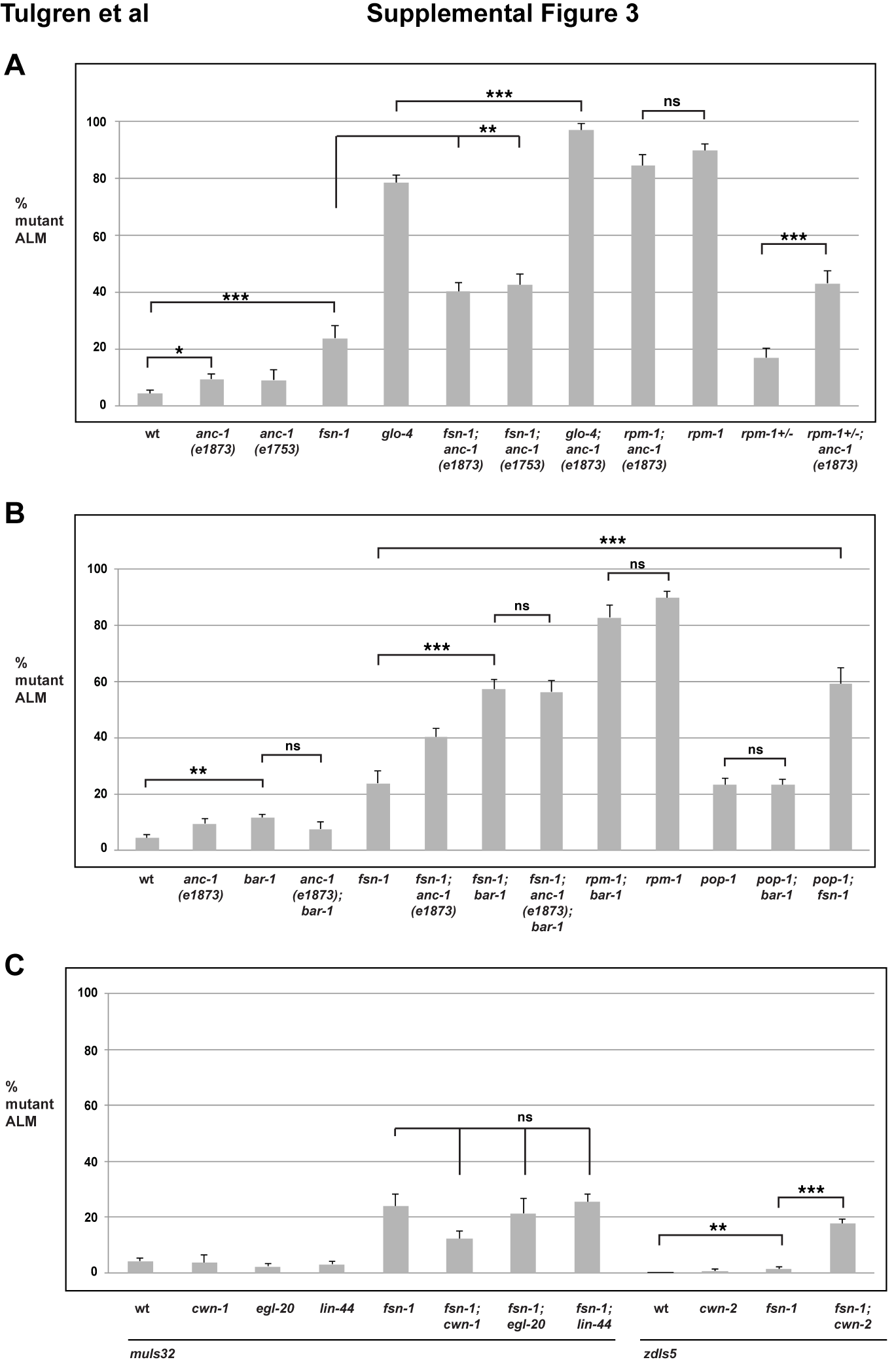

Supplement: Figure S3 — anc-1, bar-1 and the Wnt cwn-2 regulate axon termination in the ALM mechanosensory neurons. ALM axon termination defects were quantitated for the indicated genotypes using muIs32 [Pmec7GFP]. (A) anc-1 mutant analysis (B) bar-1 mutant analysis. (C) cwn-2 mutant analysis. Note that zdIs5 (Pmec-4GFP) was used for cwn-2 analysis because both muIs32 and cwn-2 are on chromosome II. Analysis was done on young adults grown at 23°C. Significance was determined using an unpaired Student's t test; error bars represent the standard error of the mean. *P<0.05, **P<0.01, ***P<0.001, ns = not significant. (TIF) [file pgen.1004481.s003.tif]

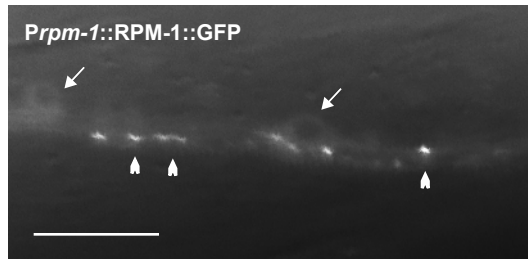

Supplement: Figure S4 — RPM-1::GFP localizes at low levels in neuronal cell bodies, but is excluded from the nucleus. Epifluorescent microscopy was used to visualize transgenic animals, juIs58, in which RPM-1::GFP was expressed using the native rpm-1 promoter. In the ventral cord motor neurons, RPM-1::GFP was concentrated at the presynaptic terminals (arrowheads). RPM-1::GFP was also localized at low levels in the cell bodies of motor neurons, where it was excluded from the nucleus (arrows). (PDF) [file pgen.1004481.s004.pdf]
